# Paxillin phosphorylation at serine 273 and its effects on Rac, Rho and adhesion dynamics

**DOI:** 10.1371/journal.pcbi.1006303

**Published:** 2018-07-05

**Authors:** Kaixi Tang, Colton G. Boudreau, Claire M. Brown, Anmar Khadra

**Affiliations:** 1 Department of Physiology, McGill University, Montreal, Québec, Canada; 2 Advanced BioImaging Facility (ABIF), McGill University, Montreal, Québec, Canada; 3 Cell Information Systems, McGill University, Montreal, Québec, Canada; 4 Department of Anatomy and Cell Biology, McGill University, Montreal, Québec, Canada; Northeastern University, UNITED STATES

## Abstract

Focal adhesions are protein complexes that anchor cells to the extracellular matrix. During migration, the growth and disassembly of these structures are spatiotemporally regulated, with new adhesions forming at the leading edge of the cell and mature adhesions disassembling at the rear. Signalling proteins and structural cytoskeletal components tightly regulate adhesion dynamics. Paxillin, an adaptor protein within adhesions, is one of these proteins. Its phosphorylation at serine 273 (S273) is crucial for maintaining fast adhesion assembly and disassembly. Paxillin is known to bind to a GIT1-βPIX-PAK1 complex, which increases the local activation of the small GTPase Rac. To understand quantitatively the behaviour of this system and how it relates to adhesion assembly/disassembly, we developed a mathematical model describing the dynamics of the small GTPases Rac and Rho as determined by paxillin S273 phosphorylation. Our model revealed that the system possesses bistability, where switching between uninduced (active Rho) and induced (active Rac) states can occur through a change in rate of paxillin phosphorylation or PAK1 activation. The bistable switch is characterized by the presence of memory, minimal change in the levels of active Rac and Rho within the induced and uninduced states, respectively, and the limited regime of monostability associated with the uninduced state. These results were validated experimentally by showing the presence of bimodality in adhesion assembly and disassembly rates, and demonstrating that Rac activity increases after treating Chinese Hamster Ovary cells with okadaic acid (a paxillin phosphatase inhibitor), followed by a modest recovery after 20 min washout. Spatial gradients of phosphorylated paxillin in a reaction-diffusion model gave rise to distinct regions of Rac and Rho activities, resembling polarization of a cell into front and rear. Perturbing several parameters of the model also revealed important insights into how signalling components upstream and downstream of paxillin phosphorylation affect dynamics.

## Author summary

Cellular migration is crucial in both physiological and pathological functions. Maintenance of proper migration and development of aberrant migration are effectuated by cellular machinery involving protein complexes, called adhesions, that anchor the cell to its environment. Over time, these adhesions assemble at the leading edge, as the cell extends forward, anchoring the front of the cells to its substrate, while those at the cell rear disassemble, allowing detachment and forward movement. Their dynamics are controlled by a number of regulatory factors, occurring on both cell-wide and adhesion-level scales. The coordination of these regulatory factors is complex, but insights about their dynamics can be gained from the use of mathematical modeling techniques which integrate many of these components together. Here, we developed several molecularly explicit models to explore how local regulation of paxillin, an adhesion protein, interacts with the activities of Rac and Rho to produce cell-wide polarization associated with motility and directionality. By altering paxillin phosphorylation/dephosphorylation within such models, we have advanced our understanding of how a shift from a non-motile state to a highly motile state occurs. Deciphering these key processes quantitatively thus helped us gain insight into the subcellular factors underlying polarity and movement.

## Introduction

In multicellular organisms, cell migration is key to proper development and maintenance of physiological processes such as embryogenesis, axonal outgrowth in neurons, and wound healing [1–5]. Additionally, aberrant migration can lead to pathological effects such as cancer metastasis [1,3–7]. To identify key factors that lead to these physiological and pathological functions, a better understanding of the biochemical regulatory pathways governing the dynamics of motility is required.

Regulation of cell migration occurs through several different mechanisms, and involves changes in protein activities that occur both globally (i.e. across the entire cell) and locally [8–11]. Polarization, for example, has historically been attributed to a cell-wide gradient in the activities of the Rho family of GTPases, including Cdc42, Rac1 (Rac), and RhoA (Rho), and their cycling between the cytoplasm and membrane binding [8,9,12–15]. Specifically, the activities of Cdc42 and Rac, known to promote actin polymerization, membrane protrusion and membrane ruffling [16–20], are thought to be high at the cell front compared to the rear, whereas the activity of RhoA, responsible for actomyosin contraction, is low at the cell front and high at the rear [8,12–14].

On a smaller scale, mechanosensitive proteins (such as talin) reside within adhesions and facilitate local regulation [21,22]. These proteins are bound to both the adhesion and the actin cytoskeleton, and can be stretched in response to actomyosin contractile force to reveal binding sites that are normally concealed under low tension [21]. Proteins such as vinculin can subsequently bind to these exposed sites and alter processes such as adhesion assembly [22]. Local regulation is not exclusively initiated by mechanosensitive proteins, however. Signalling cascades, often initiated by protein phosphorylation, may take place at adhesions without a direct dependence on tension [10,23–27]. For example, in Chinese Hamster Ovary K1 (CHO-K1) cells, p21-Activated Kinase 1 (referred to hereafter as PAK)-mediated phosphorylation of the scaffold protein paxillin at serine residue 273 (S273) increases adhesion dynamics [25] and raises the level of active Rac through the activity of a trimeric protein complex consisting of G protein-coupled receptor kinase InteracTor 1 (referred to hereafter as GIT), beta-PAK-Interacting eXchange factor (referred to hereafter as PIX), and PAK that binds to paxillin subsequent to its phosphorylation [25].

Previous studies [25] have shown that high levels of S273 phosphorylation are associated with an increase in the number of small adhesions located near the cell periphery. These adhesions assemble and disassemble relatively quickly, allowing for fast membrane protrusion and cell migration. Conversely, cells with low levels of paxillin phosphorylation exhibit relatively large adhesions with slow assembly and disassembly rates, causing cell protrusion and migration velocities to be slow as well. These results were obtained by using either phosphomimetic or nonphosphorylatable mutants of paxillin in which the serine 273 residue was replaced with aspartic acid (S273D) or alanine (S273A), respectively. In addition to phosphorylation, downregulation of paxillin dephosphorylation, through the serine/threonine phosphatase PP2A, has also been shown to increase cell spreading, motility, and metastasis [28]. Therefore, it would be interesting to investigate whether restoring levels of PP2A activity to a normal range could attenuate cancer metastases.

The combined effects of the multiple regulatory pathways involved in cell motility are thus complex and difficult to consolidate. This makes identifying key relationships challenging and predicting new relationships unintuitive. Through the use of mathematical modeling techniques, however, these processes may be integrated to explain biological phenomena, make predictions, and motivate new experimental studies.

Many quantitative models capture migration, or aspects of migration, using a balance of physical forces within the cell, including the polymerization force of actin filaments, the inward force of actomyosin contraction, and the traction force generated by cell-matrix adhesions [29–33]. Models of adhesion assembly and disassembly have been also used to predict distributions of adhesion sizes [34]. Another route of modeling has been through studying the molecular interactions of signalling proteins [13,14,33,35–39], including Cdc42, Rac, and Rho. Their interactions cause cell polarization and directed cell motion [13,14,38,39]. Analysis of these interactions revealed that polarization is achieved through bistability, a commonly observed feature in biological systems in which one of two states can be attained, depending on initial conditions, but switching between them can also occur (though a hysteresis) upon perturbations [40]. In this system, bistability results from mutual inhibition between Rac and Rho [13,14,41,42].

In similar studies, the activities of Cdc42, Rac, and Rho have also been combined with the effects of extracellular matrix-dependent protrusion velocity, contractile force, and feedback of adhesions onto Rac and Rho activities [33,35,36] to predict bistability in both the protrusion velocity and the density of stable adhesions (as determined by matrix stiffness). Variations of such models revealed that bistability persists in models of Rac and Rho that assume Rac is inactivated by Rho [37]. Using IPA-3, an inhibitor of PAK activity, the presence of bistability was confirmed experimentally [37]. Here, we employed similar modeling techniques to predict the role of paxillin S273 phosphorylation and the GIT-PIX-PAK complex in determining the dynamics and steady state behaviour of active Rac and Rho.

## Methods

### Mathematical model

#### Model assumptions and development

**Fig 1:**
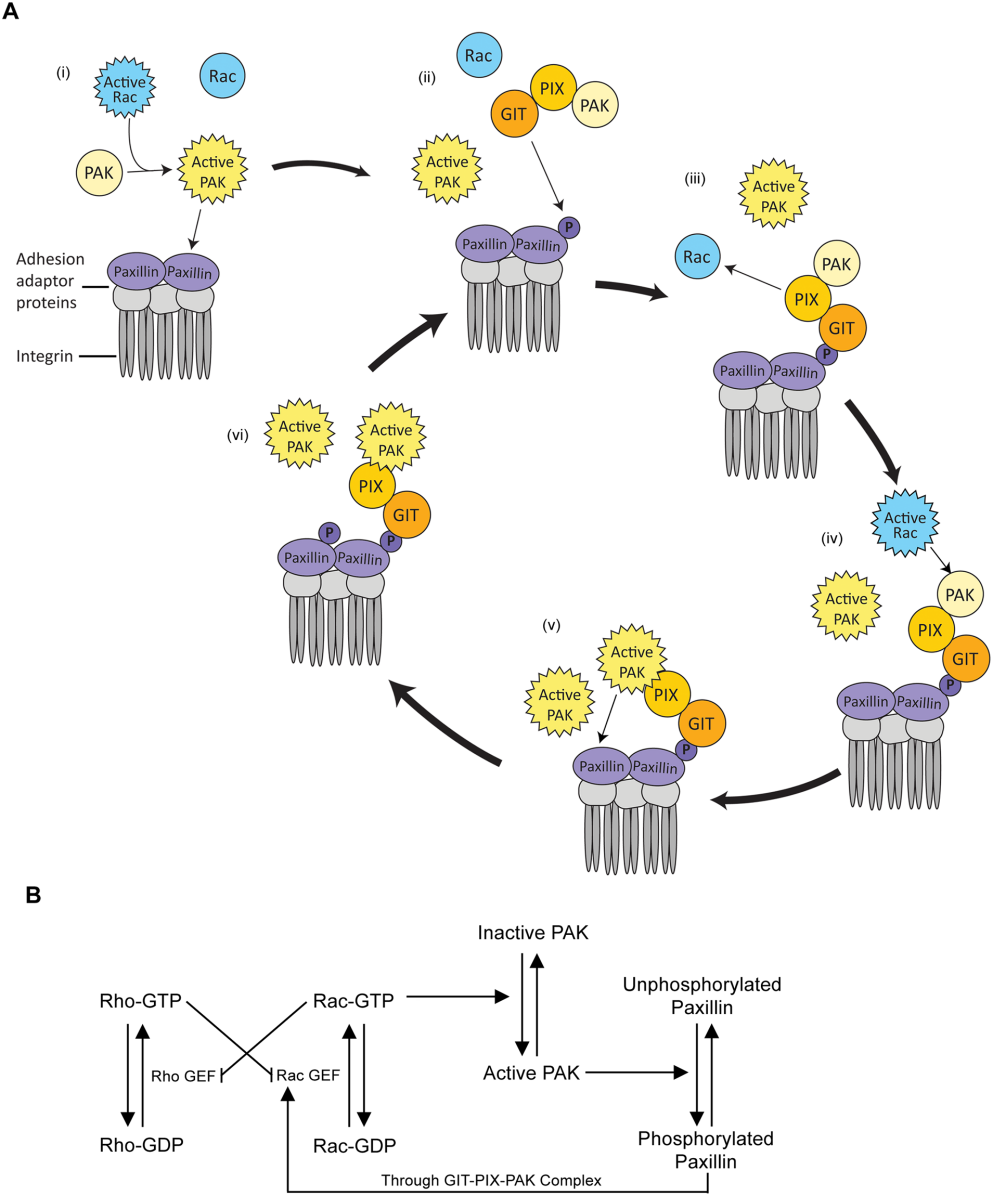
Diagram illustrating the molecular interactions of paxillin with the GIT-PIX-PAK complex and the Rac/Rho crosstalk. (A) In the cycle of paxillin dynamics, multiple steps are involved: i) Active Rac binds to and activates PAK, which in turn phosphorylates paxillin at S273. (ii) Phosphorylated paxillin, Pax_p_, binds the GIT-PIX-PAK complex. (iii) Pax_p_-bound PIX activates additional Rac proteins. (iv) Newly activated Rac phosphorylates PAK present within the GIT-PIX-PAK complex. (v) PAK present within the GIT-PIX-PAK complex phosphorylates neighbouring paxillin molecules. (vi) Phosphorylation of a neighbouring monomeric paxillin promotes its binding to GIT-PIX-PAK complexes, closing the positive feedback loop of paxillin phosphorylation, Rac activation, and PAK activation. (B) Rac and Rho both cycle between inactive (GDP-bound) and active (GTP-bound) forms, where activation occurs by GTPase-specific GEFs (including PIX), and inactivation occurs by GTPase-specific GAPs. Rac and Rho are also assumed to mutually inhibit each other through nonspecific downregulation of each other’s GTPase-specific GEFs. Rac activation/inactivation is coupled to paxillin phosphorylation by PAK activation and GIT-PIX-PAK complex formation as described by the cycle in A.

To explore the effects of paxillin phosphorylation on the levels of active Rac (RacGTP) and active Rho (RhoGTP), we constructed a model based on the relations between paxillin, the GIT-PIX-PAK complex, Rac and Rho as specified in Fig 1 (for full derivation of the model, see S1 Text). Monomeric paxillin was assumed to exist in two states: phosphorylated and unphosphorylated states (at S273). Paxillin phosphorylation occurs through the kinase activity of active PAK, where PAK is activated through binding to active Rac (i.e., all complexes containing RacGTP). Dephosphorylation is mediated by the serine/threonine phosphatase Protein Phosphatase 2A (PP2A). Once phosphorylated at S273, monomeric phosphorylated paxillin (Pax_p_) can bind to the GIT-PIX-PAK complex, and the complex-bound PAK may then be activated by active Rac to engage in subsequent paxillin phosphorylation, thus closing a positive feedback loop of PAK activation, paxillin phosphorylation, and GIT-PIX-PAK binding as suggested by Fig 1A.

Rac and Rho both exist in three forms: a membrane-bound active form associated with GTP, a membrane-bound inactive form associated with GDP, and a cytosolic inactive form [13,14]. For simplicity, we assumed that Rac and Rho exist in two states: active/membrane-bound, and inactive/unbound. This assumption is in agreement with previous evidence suggesting that once inactivated, Rac and Rho quickly dissociate from the membrane [15,43]. The activations of Rac and Rho are mediated by GTPase-specific Guanine Exchange Factors (GEFs), whereas their inactivation occurs through the activities of GTPase-specific GTPase-Activating Proteins (GAPs). Studies have suggested [8,44,45] that the active forms of Rac and Rho exert mutual inhibition on each other by downregulating each other’s GEFs (see Fig 1B). In contrast, in another model, it was hypothesized that active Rho activates Rac inactivation through GAPs instead [37]. In the model presented here, we adopted the former assumption.

The connection between paxillin S273 phosphorylation and elevated Rac activity is PIX, a known Rac-GEF which is bound to the adhesion as part of the GIT-PIX-PAK complex. As the level of S273-phosphorylated paxillin (Pax_p_) increases, so does the binding of PIX to the adhesion. The increased GEF activity that results from PIX accumulation causes a rise in the Rac activation rate.

In addition to the reactions specified above, we also made the following assumptions based on experimental evidence:

1. Rac is activated at a basal level by various Rac-GEFs, including unbound PIX, in the absence of paxillin phosphorylation. This activation rises above the basal level when paxillin S273 is phosphorylated due to the accumulation of PIX at the adhesion [25].
2. Monomeric PIX and all complexes which contain it are capable of activating Rac, since the expression of a GEF-deficient PIX mutant (capable of binding to GIT and PAK) can reverse the effects of the paxillin S273D mutant [25,46].
3. Synthesis and degradation of Rac, Rho, PAK, and paxillin occur on a much slower time scale than their binding dynamics. This means that their total concentrations are conserved [47–49]. We can quantify their total concentrations using the expression
  $$Y_{tot}=\frac{1}{L}\int_{0}^{L}(Y+Y_{i})dx,$$
  where *Y* and *Y*_*i*_, respectively, denote the total concentrations of all forms of active and inactive (or, respectively, phosphorylated and unphosphorylated) Rac, Rho, or PAK (paxillin).
4. Since GIT and PIX are more abundant in the cell than PAK [50], monomeric GIT and PIX at the cell membrane are quickly replenished from a large cytosolic pool, allowing both of their concentrations to remain constant. This assumption is required, since a constant total PIX concentration would, by Assumption 2, suggest a constant Rac activation rate associated with all three variants of paxillin (wild type and the A and D mutants).
5. The levels of the Pax_p_-GIT, Pax_p_-GIT-PIX, and GIT-PIX-PAK-RacGTP complexes are negligible, since Pax_p_-GIT binding is dependent on PIX, and both the PIX-PAK and Pax_p_-GIT interactions are required to observe the effect of paxillin S273 phosphorylation [25]. Therefore, all reactions that result in the formation of these complexes were excluded from the model.

A complete list of reactions included in the model is provided in S1 Table. In general, reactions involving binding between two proteins or protein complexes were assumed to follow mass-action kinetics. This was true for reactions involving the formation of the GIT-PIX-PAK complex and its binding to Pax_p_, as well as PAK activation and inactivation. To focus on the key variables of interest (i.e., Rac, Rho, and paxillin), the model was simplified by applying quasi-steady state (QSS) assumptions to the reactions that do not follow the activation/inactivation and phosphorylation/dephosphorylation dynamics of these proteins (i.e. reactions 3 and 5–12 in S1 Table). This allowed us to reduce the original model to a three-dimensional system expressing scaled levels of active Rho (*ρ*), active Rac (R*), and phosphorylated paxillin S273, present in both monomeric and complex forms (referred to hereafter as phosphopaxillin, P*).

#### Model framework

As a result of the assumptions listed above, the collective concentration of active PAK, in all of its complex-bound forms, is at steady state and may be expressed as

$$\left[PAK^{*}]=[PAK-RacGTP]+[PIX-PAK-RacGTP]+[Pax_{p}-GIT-PIX-PAK-RacGTP]=\frac{k_{PAK}^{+}}{k_{PAK}^{-}}[PAK][RacGTP](1+\frac{k_{X}^{+}}{k_{X}^{-}}[PIX]+\frac{k_{G}^{+}k_{X}^{+}k_{C}^{+}}{k_{G}^{-}k_{X}^{-}k_{C}^{-}}[Pax_{p}][GIT][PIX]\right)$$

The dynamics of Rac were modeled based on a previously established work [13,14]. Specifically, the rate of Rac inactivation was assumed to follow mass-action kinetics with a rate constant *δ*_*R*_, whereas the activation rate (which depends on the level of bound PIX), was assumed to be proportional to the sum of two rates: $I_{R}+I_{K}^{*}$, where *I*_*R*_ is a constant basal rate of activation (that depends on the level of monomeric PIX and GIT-PIX only) and $I_{K}^{*}$ is the rate that depends on the level of the remaining complex-bound PIX. Crosstalk (seen in Fig 1B) was modeled as a cooperative reduction of the activation rate, and was expressed using a Hill function dependent on the concentration of active Rho (i.e., RhoGTP), with a Hill coefficient *n* and a half-maximal inhibition *L*_*Rho*_. Rescaling the concentrations of Rac and Rho to their total concentrations, we obtain

$$R=\frac{\left[RacGTP\right]}{\left[Rac_{tot}\right]}$$

$$R_{i}=\frac{\left[RacGDP\right]}{\left[Rac_{tot}\right]}$$

$$\rho =\frac{\left[RhoGTP\right]}{\left[Rho_{tot}\right]}$$

$$\rho_{i}=\frac{\left[RhoGDP\right]}{\left[Rho_{tot}\right]}.$$

The dynamics of scaled RacGTP and RacGDP are thus given by (see S1 Text)

$$\frac{\partial R}{\partial t}=(I_{R}+I_{K}^{*})(\frac{L_{\rho }^{n}}{L_{\rho }^{n}+\rho^{n}})R_{i}-\delta_{R}R+D_{R}\frac{\partial^{2}R}{\partial x^{2}}$$ (1)

$$\frac{\partial R_{i}}{\partial t}=-(I_{R}+I_{K}^{*})(\frac{L_{\rho }^{n}}{L_{\rho }^{n}+\rho^{n}})R_{i}+\delta_{R}R+D_{R_{i}}\frac{\partial^{2}R_{i}}{\partial x^{2}},$$ (2)

where $L_{\rho }=\frac{L_{Rho}}{\left[Rho_{tot}\right]}$. The rate $I_{K}^{*}$ can be expressed as

$$I_{K}^{*}=I_{K}^{'}(\left[PIX-PAK]+[GIT-PIX-PAK]+[Pax_{p}-GIT-PIX-PAK]+[PIX-PAK-RacGTP]+[Pax_{p}-GIT-PIX-PAK-RacGTP\right]),$$

where $I_{K}^{\prime }$ is the PIX-mediated rate of Rac activation. After substituting the steady state expressions for each intermediate species and substituting the rescaled rate parameter $I_{K}=I_{K}^{\prime }[PAK_{tot}]$, the expression for $I_{K}^{*}$ becomes

$${I_{K}^{*}=I}_{K}(1-K_{i}^{*}(1+\alpha_{R}R)),$$

where $K_{i}^{*}$, the concentration of monomeric PAK relative to the total PAK concentration, is expressed as

$$K_{i}^{*}={\left\{\left(1+k_{X}[PIX]+k_{G}k_{X}k_{C}[GIT][PIX][Pax_{tot}]P)(1+\alpha_{R}R)+k_{G}k_{X}[GIT][PIX\right]\right\}}^{-1}.$$

The dynamics of Rho were modeled similarly, i.e., both the activation and inactivation were assumed to occur with rate constants *I*_*ρ*_ and *δ*_*ρ*_, respectively. Inhibition of Rho activation, in the presence of crosstalk, was also modeled as a Hill function that depends on the collective concentration of active Rac (i.e. RacGTP and all complexes bound to it), with a Hill coefficient *n* and a half-maximal inhibition *L*_*Rac*_. The equations describing the dynamics of Rho are thus given by

$$\frac{\partial \rho }{\partial t}=I_{\rho }(\frac{L_{R}^{n}}{L_{R}^{n}+{\left(R+\gamma K\right)}^{n}})\rho_{i}-\delta_{\rho }\rho +D_{\rho }\frac{\partial^{2}\rho }{\partial x^{2}}$$ (3)

$$\frac{\partial \rho_{i}}{\partial t}={-I}_{\rho }(\frac{L_{R}^{n}}{L_{R}^{n}+{\left(R+\gamma K\right)}^{n}})\rho_{i}+\delta_{\rho }\rho +D_{\rho_{i}}\frac{\partial^{2}\rho_{i}}{\partial x^{2}}.$$ (4)

The reactions governing the dynamics of paxillin are phosphorylation, dephosphorylation, and binding to and unbinding from the GIT-PIX-PAK complex. The latter two (described by Reaction 11 in S1 Table) were set to steady state, leaving only phosphorylation and dephosphorylation. Since phosphorylation occurs through a transient binding between active PAK (i.e., PAK*) and paxillin, the rate of paxillin phosphorylation was modeled as a Hill function that increases with the scaled concentration of active PAK, *K*, defined by $K=\frac{\left[PAK^{*}\right]}{\left[PAK_{tot}\right]}$. This Hill function depends on the Hill coefficient *n*, the maximum phosphorylation rate *B*, and the scaled half-maximum phosphorylation *L*_*K*_. Dephosphorylation, on the other hand, was assumed to follow mass-action kinetics proportional to the rate constant *δ*_*P*_ that depends on the concentration of PP2A (assumed to be constant). Therefore, the dynamics of phosphorylated and unphosphorylated paxillin (*P* and *P*_*i*_, respectively), scaled by the total paxillin concentration *Pax*_*tot*_, are given by

$$\frac{\partial P}{\partial t}=B(\frac{K^{n}}{L_{K}^{n}+K^{n}})P_{i}-\delta_{P}P+D_{P}\frac{\partial^{2}P}{\partial x^{2}}$$ (5)

$$\frac{\partial P_{i}}{\partial t}=-B(\frac{K^{n}}{L_{K}^{n}+K^{n}})P_{i}+\delta_{P}P+D_{P_{i}}\frac{\partial^{2}P_{i}}{\partial x^{2}}.$$ (6)

The spatiotemporal (reaction-diffusion) model given by Eqs (1)–(6) was used to study polarization in active Rac and active Rho.

#### Parameter estimation

Parameter estimation was done by fitting different aspects of the model to previously published experimental data obtained from the literature. A summary of the estimated parameter values is provided in Table 1. Note that in [51], 1D experimental geometry was estimated over the width and depth of the cell, but these have been accounted for in our parameter estimations. See S1 Text for more details about the full derivations of parameter values.

**Table 1:** Summary of parameter estimations.

Parameter	Description	Value	Unit	References
*L*	Cell length	10	μm	[14]
*I*_*ρ*_	Rho activation rate	0.016	s^-1^	[52,53]
*δ*_*ρ*_	Rho inactivation rate	0.016	s^-1^	[52]
*L*_*R*_	Rho level at half-maximal inhibition	0.34	Unitless	[52–54]
*I*_*R*_	Basal Rac activation rate	0.003	s^-1^	Estimated
*I*_*K*_	Additional Rac activation rate	0.009	s^-1^	Estimated
*L*_*ρ*_	Rac level at half-maximal inhibition	0.34	Unitless	[52–54]
*δ*_*R*_	Rac inactivation rate	0.025	s^-1^	Estimated
*γ*	Ratio of total PAK to total Rac	0.3	Unitless	Estimated
*α*_*R*_	Affinity constant for PAK-RacGTP binding	15	Unitless	[13,14,55]
*k*_*G*_	Association constant for GIT-PIX binding	5.71	s^-1^	[56]
*k*_*X*_	Association constant for PIX-PAK binding	41.7	s^-1^	[46]
*k*_*C*_	Association constant for Pax_p_-GIT binding	5	s^-1^	Estimated
*B*	Maximum paxillin- phosphorylation rate	4.26	s^-1^	[57]
*L*_*K*_	Scaled level of paxillin at half-maximum activation	5.77	Unitless	[57]
*δ*_*P*_	Paxillin dephosphorylation rate	0.00041	s^-1^	[57]
*n*	Hill coefficient; level of cooperativity	4	unitless	[13,14]
[*GIT*]	Concentration of GIT	0.11	μM	[50,58]
[*PIX*]	Concentration of PIX	0.069	μM	[50,58]
[*Pax*_*tot*_]	Total concentration of paxillin	2.3	μM	[50,58]
*D*_*ρ*_	Diffusion coefficient of active Rho	0.02	μm^2^/s	[59]
$D_{\rho_{i}}$	Diffusion coefficient of inactive Rho	0.43	μm^2^/s	[59]
*D*_*R*_	Diffusion coefficient of active Rac	0.02	μm^2^/s	[59]
$D_{R_{i}}$	Diffusion coefficient of inactive Rac	0.43	μm^2^/s	[59]
*D*_*P*_	Diffusion coefficient of phosphorylated paxillin	0.03	μm^2^/s	[60,61]
$D_{P_{i}}$	Diffusion coefficient of unphosphorylated paxillin	0.03	μm^2^/s	[60,61]

#### Model simplification

To analyze the steady state behaviour of the spatiotemporal model of Eqs (1)–(6), we first assumed that the diffusion coefficients in these equations are equal to 0. Local perturbation analysis (LPA) [62] was not applied here because the diffusion coefficients of the different molecular species under consideration do not differ significantly. Based on Assumption 3 above, we can conclude that the total concentrations of Rho, Rac, paxillin, and PAK are constant. These total concentrations are given by

$$\begin{array}{l} \left[Rho_{tot}\right]=\left[RhoGDP\right]+\left[RhoGTP\right]\\ \left[Rac_{tot}\right]=\left[RacGDP\right]+\left[RacGTP\right]+\left[PAK-RacGTP\right]+\left[PIX-PAK-RacGTP\right]+\left[Pax_{p}-GIT-PIX-PAK-RacGTP\right]\\ \left[Pax_{tot}\right]=\left[Pax\right]+\left[Pax_{p}\right]+\left[Pax_{p}-GIT-PIX-PAK\right]+\left[Pax_{p}-GIT-PIX-PAK-RacGTP\right]\\ \left[PAK_{tot}\right]=\left[PAK\right]+\left[PIX-PAK\right]+\left[GIT-PIX-PAK\right]+\left[Pax_{p}-GIT-PIX-PAK\right]+\left[PAK-RacGTP\right]+\left[PIX-PAK-RacGTP\right]\\ +\left[Pax_{p}-GIT-PIX-PAK-RacGTP\right]. \end{array}$$

This allowed us (see S1 Text) to reduce the resulting six-dimensional ODE model to the following three-dimensional system

$$\frac{d\rho }{dt}=I_{\rho }(\frac{L_{R}^{n}}{L_{R}^{n}+{\left(R+\gamma K\right)}^{n}})(1-\rho )-\delta_{\rho }\rho$$ (7)

$$\frac{dR}{dt}=(I_{R}+I_{K}^{*})(\frac{L_{\rho }^{n}}{L_{\rho }^{n}+\rho^{n}})(1-R-\gamma K)-\delta_{R}R$$ (8)

$$\frac{dP}{dt}=B(\frac{K^{n}}{L_{K}^{n}+K^{n}})(1-P(1+k_{G}k_{X}k_{C}[GIT][PIX][PAK_{tot}]K_{i}^{*}(1+\alpha_{R}R)))-\delta_{P}P.$$ (9)

Eqs (7)–(9) were used to examine the scaled steady state levels of RhoGTP (i.e., active Rho), RacGTP, and Pax_p_. For the latter two species, we extended our analysis to both monomeric and complex-bound forms of the two proteins, which we distinguished from their monomeric concentrations by denoting them as R* for active Rac and P* for phosphopaxillin, given by

$$R^{*}=\frac{\left[RacGTP]+[PAK-RacGTP]+[PIX-PAK-RacGTP]+[Pax_{p}-GIT-PIX-PAK-RacGTP\right]}{\left[Rac_{tot}\right]}$$ (10)

and

$$P^{*}=\frac{\left[Pax_{p}]+[Pax_{p}-GIT-PIX-PAK]+[Pax_{p}-GIT-PIX-PAK-RacGTP\right]}{\left[Pax_{tot}\right]}.$$ (11)

The *ρ*-, *R*- and *P*-nullsurfaces associated with Eqs (7)–(9), respectively, may be obtained by setting these three equations to zero. One can then use Eqs (10) and (11) (along with the steady states expression for the other intermediates) to define the R*- and P*-nullsurfaces derived from the *R*- and *P*-nullsurfaces, respectively. Then, the intersections of the *ρ*-nullsurface (dependent on R*) and R*-nullsurface (dependent on *ρ* and P*) can be projected onto the P*-R* plane along with the projected P*-nullsurface (dependent on R*). In our analysis, we used *ρ*, R* and P* variables and their corresponding nullsurfaces to investigate this system. We also used them to study the spatiotemporal dynamics of Eqs (1)–(6), in which *ρ*, R* and P* (along with the steady state expressions of intermediates and complexes) were calculated from *ρ*, *ρ*_*i*_, *R*, *R*_*i*_, *P* and *P*_*i*_.

### Experimental methods

#### Cell culture and transfection

Chinese Hamster Ovary K1 (CHO-K1) cells (Sigma Aldrich, 85050302) were grown with low-glucose DMEM containing the following: L-glutamine, 100 mg/L sodium pyruvate, pyridoxine hydrochloride (ThermoFisher Scientific, Grand Island, NY, 11885–084), 10% vol/vol Fetal Bovine Serum (ThermoFisher Scientific, 10082–147), 1% vol/vol 100X non-essential amino acids (ThermoFisher Scientific, 11140–050), 10,000 units/mL penicillin/10,000 μg/mL streptomycin (ThermoFisher Scientific, 10378–015) and 25 mM HEPES (Sigma Aldrich, H0887). Cells were split every 2–3 days as follows. Cells were washed once with 5 mL of warm phosphate buffered saline (PBS) and lifted with 500 μL of trypsin/EDTA for 5 min. Trypsin was then neutralized with 500 μL culture media. After cells were suspended, they were placed into new culture plates containing 5 mL of culture media and incubated at 37°C in an incubator with an environment of 5% CO_2_ balanced with air.

To transfect cells with the Rac1-2G, approximately 75,000 cells were seeded into 6 well plates after suspending cultured cells with trypsin/EDTA. Seeded cells were grown overnight and transfected the following day with 0.5 μg of Rac1-2G in 1.25 μL Lipofectamine 2000 (ThermoFisher Scientific, 11668–027) and incubated overnight before imaging. To obtain FRET reference spectra, control samples were transfected with 4 μg of mTFP or Venus alone using 10 μL Lipfectamine 2000. mTFP1-pBAD was a gift from Robert Campbell & Michael Davidson (Addgene plasmid # 54553), and Venus-pBAD was a gift from Michael Davidson (Addgene plasmid # 54859). pTriExRhoA2G and pTriEx4-Rac1-2G were gifts from Olivier Pertz (Addgene plasmid # 40176 and # 66110, respectively). Paxillin-EGFP, S273D and S273A plasmids were a gift from Rick Horwitz (University of Virginia).

#### Live cell tracking and migration speeds

Paxillin WT or PIX-ΔGBD cells were plated on fibronectin coated μ-Slide 8 Well imaging slides (ibidi, Cat#80826). Cells were incubated for 2–3 hours and then placed in a microscope stage top environmental control chamber (Live Cell Instrument, Seoul, Korea) and maintained at 37°C under a 5% CO_2_ humidified environment with a flow rate of 50 mL/min. The chamber was placed on the stage of an inverted microscope (Axiovert 200M, Carl Zeiss) with an Axiocam 506 monochrome camera (Zeiss) and 20x objective (NA 0.5, Zeiss). Phase contrast transmitted light imaging with exposure times of 150–300 ms were used to acquire timelapse images. A multi-dimension acquisition was programmed using AxioVision 4.8.2 software, where five sites per well were chosen and imaged every 10 minutes for a total of 18 hours.

Cell tracking data was obtained by loading the sequential images as a stack in MetaXpress 5.0 (Molecular Devices Inc., Sunnyvale, CA) and semi-manual cell tracking was carried out using the “*track points*” function. A minimum of 45 cells were tracked for each experiment for a minimum total track length of 6 hours. The data was logged into Microsoft Excel and average cell speeds were calculated by dividing the distance moved between frames by 1/6 of an hour. The average speed of each cell was calculated by averaging the instantaneous speed between each frame tracked over the six-hour period.

#### Live cell imaging conditions

To image transfected cells, 35 mm glass bottom Fluorodishes (World Precision Instruments, Sarasota, FL, FD35) with a 0.17 μm thick coverslip bottom were coated using 2 μg/mL fibronectin (Sigma Aldrich, F0985) in PBS for 1 hour at 37°C under 5% CO_2_. Plates were then washed twice with warm PBS. The transfected cells were lifted with 250 μL of trypsin/EDTA for 5 min. Trypsin was then neutralized with 750 μL culture media. Approximately 25,000 of the suspended cells were plated on the glass bottom dishes in 2 mL of cell culture media and incubated at 37°C with 5% CO_2_ for 24 hours before imaging.

#### Live cell TIRF imaging and quantification of adhesion assembly/disassembly rates and sizes

Live cell Total Internal Reflection Fluorescence (TIRF) microscopy was performed by placing a glass bottom tissue culture plate in a microscope stage top live cell environmental control chamber (Live Cell Instrument, CU-501) to keep the cells at 37°C with 5% CO_2_ flowing at 50 mL/min. TIRF microscopy was achieved using a prism-based Spectral Diskovery system (Spectral Applied Research, Richmond Hill, ON) attached to an inverted DMI6000 microscope (Leica Microsystems, Wetzler, Germany). Images were taken with a Leica Plan Apochromate 63x/1.47 NA TIRF oil immersion objective lens, using a 488 nm diode laser for excitation and an ImagEMX2 Digital EM-CCD camera (Hamamatsu, Hamamatsu City, Japan). To view samples using the eyepieces, a 100-watt X-Cite 120LED (370–700 nm) light source (Excelitas Technologies, Waltham, MA) was used along with an EGFP filter cube (ET 424/40 nm). MetaMorph (Molecular Devices Inc.) acquisition software and custom-designed TIRF controls (Quorum Technologies Inc., Guelph, ON) were used.

To image samples, cells were first located with the eyepieces. Samples were excited with 488 nm laser at 10% power with 10% laser pulsing using the Quorum Flicker device to pulse the light, resulting in a net power of 1%. Images were captured every 30 seconds for 30 min at 80 nm TIRF penetration depth, with exposure times of 300 ms, a camera read speed of 11 MHz, and an EM gain of 100 out of 255.

Acquired images were background subtracted in MetaXpresss 5.0 by selecting a region in the image with no cells, measuring the average intensity and using the statistical background correction function to subtract this value from each pixel in the frame. For each time lapse image, adhesions were identified and tracked over time using Imaris 7.5.2 (Andor Technology, Belfast, Ireland) by creating a surface for each time lapse image and setting the parameters for using the Surface Creation Wizard as follows. First, a region of interest containing only the cell of interest was selected. To track the surfaces, the “Track Surfaces (over Time)” box was selected in the Creation wizard. Before the surfaces were computed, a Gaussian filter was applied with the “Smooth” option selected and the “Surface Area Detailed Level” set to 0.25 μm. The “Background Subtraction” option was chosen for the surface creation threshold to apply a local background subtraction to the image by applying a Gaussian filter to the image and subtracting the filter from every pixel of the image. The “Sphere Diameter”, or the maximum sphere which fits into an adhesion, was set to 0.25 μm. After the surfaces were computed, background pixels which were detected as adhesions were removed by manually setting an intensity threshold so that only adhesions were selected for further analysis. To split touching adhesions, the “Split touching Objects” option was selected with a “Seed Points Diameter” of 0.75 μm. This sets the minimum size of an adhesion. The seed points were then filtered by manually setting a surface area threshold such that each adhesion was associated with one object. The remaining seed points were then converted back into surfaces, and any filters for these surfaces were deleted. Finally, to track the surfaces, the “Autoregressive Motion” algorithm was selected with a “Maximum Distance” of 2 μm to set an upper limit on the distance an adhesion could travel from one frame to the next. The “Maximum Gap Size” was set to 3 to impose a maximum number of time frames where an adhesion was not detected before tracking of its lineage stopped. The resulting adhesion area and mean intensity data were then exported to .csv files and either imported into Microsoft Excel or MATLAB (MathWorks, Natick, MA).

To determine adhesion assembly and disassembly rates, the mean intensity of each adhesion was plotted over time. Phases of assembly and disassembly were identified manually by determining if the fluorescence intensity was decreasing or increasing, respectively. The assembly rate was determined as the slope of the graph of ln(intensity/initial intensity). The disassembly rate was determined as the slope of the graph of ln(initial intensity/intensity).

#### FRET imaging and quantification

During imaging, samples were placed in a live cell environmental control chamber (Live Cell Instrument, CU-501) kept at 37°C with 5% CO_2_ flowing at 50 mL/min. Samples were viewed through the eyepiece with a 100 watt X-Cite 120LED (370–700 nm) light source (Excelitas Technologies, Waltham, MA) and an Zeiss FS38 (eGFP) filter cube. Spectral image acquisition was performed on a Zeiss 710 confocal laser scanning microscope (Jena, Germany) with a Quasar 32 photomultiplier tube array detector, using Zen Software to detect wavelengths from 415 nm to 650 nm. mTFP and Venus reference spectra were obtained as described by steps 70–72 of [63]. The following imaging settings were used for experimental spectral images: magnification, 63x/1.4 NA; wavelengths collected, 415 to 650 nm; zoom, 2x; bit depth, 12-bit; image dimensions, 1024x1024 pixels; scan speed, 5 (pixel dwell, 6.30 μs); line averaging, 8; detector gain, 650; digital gain, 1; offset, 20; pinhole, 4.01 Airy units, 405 nm laser power, 7%.

Okadaic acid (OA) was obtained from Sigma Aldrich (O7760) and dissolved in ddH_2_O. Before treatment, cells were imaged to obtain images at 0 nM OA. Cells were then treated with 20 nM OA for 20 min at 37°C with 5% CO_2_ before imaging for the 20 nM condition. After imaging, samples were washed three times with media and incubated at 37°C and 5% CO_2_ for 20 min before imaging for the washout condition.

After acquiring images, autofluorescence, TFP, and Venus channels were separated from images with the Linear Unmixing algorithm in the Zen software using the autofluorescence, TFP, and Venus reference spectra acquired as described above. Image FRET ratios were calculated using a MATLAB script that performed the following calculations. Images were first corrected for background fluorescence as described in steps 50–53 of [63]. The corrected images were multiplied with a binary cell mask. The cell mask was created using the maximum intensity projection of the spectral (32-stack) images, using a threshold determined by the MATLAB function *graythresh*. Pixel-by-pixel FRET ratios were finally calculated using Eq (2) of [63], where *FRET*_*Corr*_ refers to the background- and crosstalk-corrected FRET image, and *CFP* refers to the image obtained from the CFP channel.

To determine the distributions of FRET ratios, the mean FRET ratio of each cell was calculated, and the distribution of mean FRET ratio across 20–31 cells were determined for each condition. The percentage of cells with high FRET ratios was calculated as the percentage of cells with FRET ratios higher than the mode of the distribution in the untreated cells.

### Software

Western blot quantification was performed using Fiji [64]. Numerical simulations and bifurcation analysis were done using MATLAB (MathWorks, Natick, MA) and xppauto (a freeware available at http://www.math.pitt.edu/~bard/xpp/xpp.html). Data were digitized with WebPlotDigitizer (Ankit Rohatgi, Austin, TX, available at https://automeris.io/WebPlotDigitizer). Steady state expressions for intermediate complexes (see S1 Text) were derived using Wolfram Mathematica (Wolfram, Champaign, IL).

## Results

### Bimodality in adhesion dynamics in wild type cells is due to bistability dictated by the initial level of phosphopaxillin

**Fig 2:**
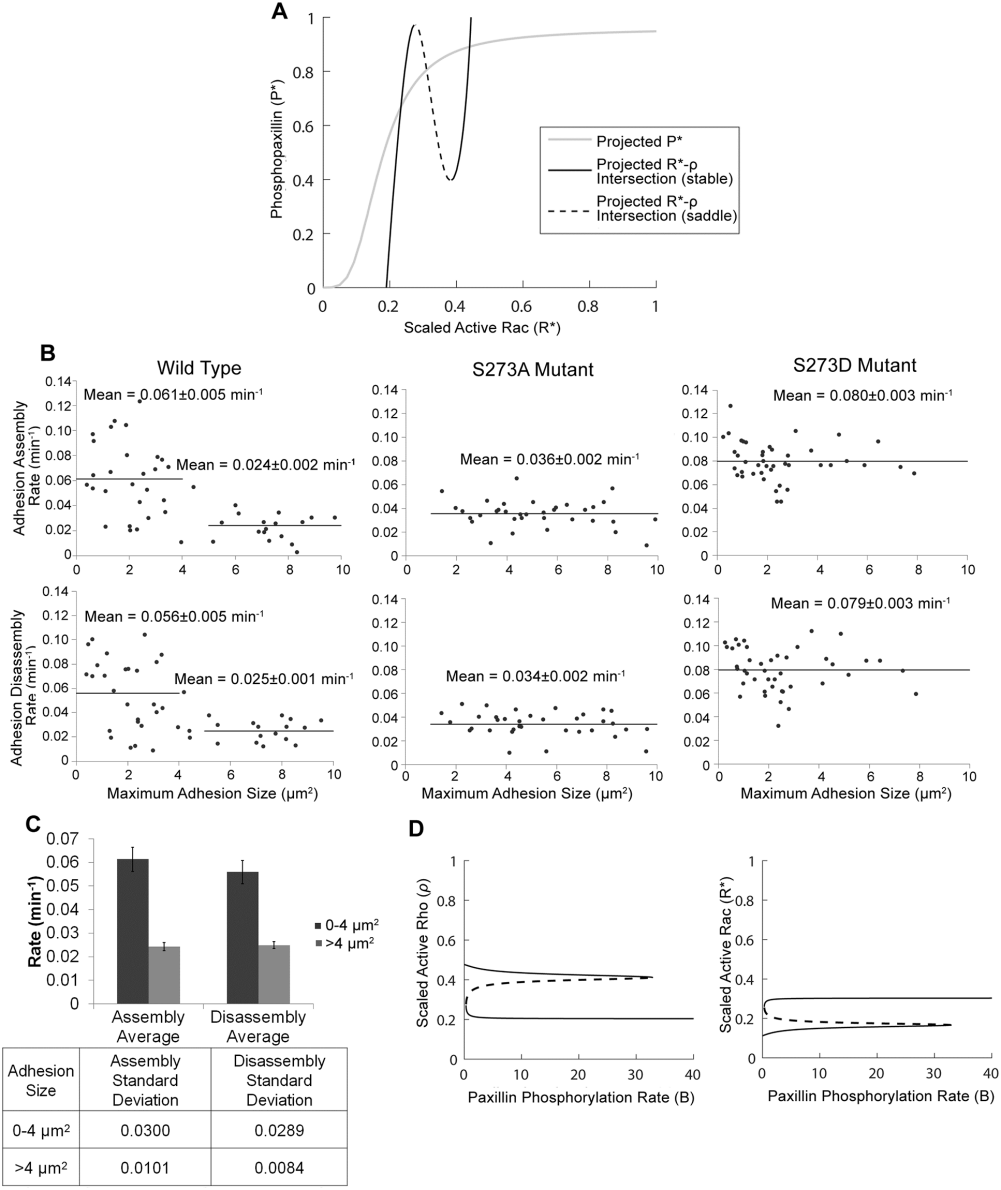
The effects of initial level of phosphorylated paxillin (P*) and maximum paxillin-phosphorylation rate (*B*). (A) Black (solid and dashed) lines represent the projected intersections of the active Rho *ρ*- and active Rac R*-nullsurfaces on the P*- R* plane, while gray line represents the projection of the P*-nullsurface on the same plane. The intersections of the solid (dashed) black lines (line) with the gray line represent the projected stable steady states (saddle). The slope of the dashed back line determines the sensitivity of the model to perturbations in the initial value of P*. Specifically, if the gray line is inclined, and the initial values of *ρ* and R* are chosen to be fixed near the dashed line of the P*-R* plane, then the system will be sensitive to vertical changes in the initial value of P*. (B) Adhesion assembly (top row) and disassembly (bottom row) rates in CHO-K1 cells expressing WT-paxillin (left panels), paxillin S273A mutant (middle panels), or paxillin S273D mutant (right panels), plotted in terms of maximum adhesion size. Cells expressing WT-paxillin show two subpopulations of adhesions exhibiting distinct assembly and disassembly rates: slow assembly/disassembly for large adhesions and fast assembly/disassembly for small adhesions. Cells expressing either S273A or S273D mutant have only one of these two populations: slow or fast, respectively. (C) Mean ± standard deviation (upper) along with the standard deviation (lower) of assembly and disassembly rates of adhesions by size in CHO-K1 cells expressing WT paxillin-EGFP. Two asterisks (**) indicate p < 0.01 by the two-tailed, unequal variance t-test. (D) Bifurcation diagrams of *ρ* (left) and R* (right) with respect to *B*, showing the steady state levels of these two variables in the induced (elevated R*) and uninduced (elevated *ρ*) states; solid lines represent stable steady states, dashed lines represent saddle points. Bistability persists for *B* ∈ (0.3,32.7), whereas monostable regimes of uninduced and induced states lie to the left and right of the bistable regime, respectively.

It was previously shown with CHO-K1 cells that the D and A mutants of paxillin S273 are associated with more dynamic or more stable adhesions and more motility or less motility, respectively, whereas cells expressing wild type paxillin exhibited features that are intermediate to these two mutants [25]. To first determine whether the initial level of phosphopaxillin is a factor in reproducing the phenotype associated with wild type cells at steady state, we plotted the projected intersections of the *ρ*- and R*-nullsurfaces (black lines) along with the projected P*-nullsurface (gray line), onto the P*-R*-plane (Fig 2A). The three intersections between the two resulting curves mark the projected steady states of the system onto the same plane. When the binding between Pax_p_ and the GIT-PIX-PAK complex (defined by the rate constant *k*_*C*_) was sufficiently large (= 5 s^-1^), the initial value of P* played a crucial role in determining the long term behaviour of *ρ* and R*. Fig 2A shows that there was a range of initial values of R* where a low initial P* value (to the left of the dashed black line) would cause R* to converge to its lower steady state (referred to hereafter as the uninduced state), and a higher initial P* value (roughly to the right of the dashed black line) that would cause R* to converge toward the elevated steady state (referred to hereafter as the induced state). In other words, the middle steady state is a saddle whose stable manifold acts as a separatrix (boundary) demarcating the basins of attraction of the induced and uninduced states. These results suggest that, depending on the initial level of phosphopaxillin ($P_{0}^{*}$), the system may converge to one of two steady state levels of active Rac: reduced or elevated, in a phenomenon referred to as bistability. It also shows that the slope of the stable manifold of the saddle (which happens to be close to the dashed black line) determines how sensitive the system is to perturbations in $P_{0}^{*}$.

To experimentally verify whether bistability in the expression level of active Rac and Rho is present within the cell, we examined the assembly and disassembly rates of adhesions, which are regulated in part by the activities of Rac and Rho, in wild type CHO-K1 cells or in cell lines overexpressing one of the two mutant paxillin proteins (see Fig 2B). In cells expressing wild type paxillin (left panels), there were two subpopulations of adhesions that appeared to exist, one with smaller more dynamic adhesions with high rates of assembly (top)/disassembly (bottom), and another with larger more stable adhesions with slower rates of assembly (top)/disassembly (bottom). In contrast, both the A (middle panels) and D (right panels) mutants exhibited only one population of adhesions. In the A mutant, all adhesions assembled (top)/disassembled (bottom) slowly, whereas in the D mutant, all adhesions assembled (top)/disassembled (bottom) quickly regardless of adhesion size. For the list of all assembly and disassembly rates, see Table 2. This suggests that in either case, one population of adhesions is conserved while the other is absent. The existence of the two subpopulations of adhesions in wild type cells, with significantly different assembly/disassembly rates (Fig 2C), is indicative of the presence of two distinct levels of Rac and Rho activities, where a state with high Rac and low Rho activity would be capable of inducing fast adhesion assembly/disassembly rates, whereas a state with low Rac and high Rho activity would cause slow adhesion assembly/disassembly rates to occur. Together with our model, these experimental results suggest that, in wild type paxillin-expressing cells, an intermediate range of initial levels of phosphopaxillin within the cells, can lead to both (i) high Rac/low Rho activity causing fast adhesion assembly/disassembly, and to (ii) low Rac/high Rho activity leading to slow adhesion assembly/disassembly. In other words, there is a mixture of adhesion assembly/disassembly rates in these wild type cells. Interestingly, the disappearance of one of the two subpopulations of adhesions in the A and D mutants also indicates that paxillin phosphorylation rate plays a crucial role in regulating bistability.

**Table 2:** Mean ± std of assembly and disassembly rates in adhesion populations present in CHO-K1 cells expressing wild type, S273A, or S273D paxillin.

	Wild Type	S273A Mutant	S273D Mutant
Assembly Rate(s) (min^-1^)	Fast: 0.061 ± 0.005	0.036 ± 0.002	0.080 ± 0.003
	Slow: 0.024 ± 0.002		
Disassembly Rate(s) (min^-1^)	Fast: 0.056 ± 0.005	0.034 ± 0.002	0.079 ± 0.003
	Slow: 0.025 ± 0.001		

### Bistability with respect to paxillin phosphorylation (*B*) or dephosphorylation (*δ*_*P*_) rates

In summary, we have shown that Rac/Rho activity depends on both the initial level of phosphopaxillin in cells expressing wild type paxillin and on paxillin phosphorylation rate in the A and D mutants. In this section, we studied with our model how varying the rates of paxillin S273 phosphorylation (*B*) and dephosphorylation (*δ*_*P*_) rates (see Eq (9) in Methods Section) affect Rac and Rho activities.

#### Effects of maximum paxillin-phosphorylation rate B

We began first by analysing the effects of the paxillin S273 phosphorylation rate on the dynamics of the system using bifurcation analysis. The steady state level of a specific protein was plotted as a function of a given parameter. Fig 2D shows that the bifurcation diagrams of active Rho, *ρ* (left) and active Rac, R* (right), with respect to *B*, the maximum paxillin-phosphorylation rate, exhibited bistability. The bistability was manifested in the plots as two solid lines, representing the stable induced and uninduced steady states, separated by a dashed line of saddle points for values of *B* ∈ (0.3,32.7). For values of *B* outside this interval, the system was monostable: only one line of uninduced (induced) steady states existed to the left (right) of the bistable regime. The transition from bistability to monostability occurred at saddle node bifurcations, where one solid line of stable steady states merged with the dashed line of saddle points and disappeared, leaving only the other solid line of stable steady states beyond the saddle node. From these results, we can conclude that a sufficiently large change in the value of *B* can trigger switching between a high *ρ*/low R* state and vice versa. That is, an increase in *B* past the right saddle node can cause the system to switch from the uninduced to the induced state, while a decrease beyond the left saddle node can cause a switch in the opposite direction. The presence of such bistability over an extended range of *B* means that it is possible to switch the system from the uninduced state, starting from a specific value of *B* ∈ (0.3,32.7), into the induced state by increasing *B* past the right saddle node but not recover upon decreasing *B* back to its original value (producing what we call memory).

The two main features of the bistable switch displayed in Fig 2D are that (i) the levels of active Rho *ρ* (left) and active Rac R* (right) do not change significantly within the uninduced and induced states (solid lines), and (ii) the range of monostability associated with the uninduced state is very small (for *B* ∈ [0,0.3)) which implies that the transition from the induced to the uninduced state is more tenable than switching in the opposite direction. These two outcomes appear to be in agreement with the observations made in [37] that after inhibiting PAK activity (and the production of new active Rac molecules) by adding increasing concentrations of IPA-3, the level of active Rac remained persistently semi-plateaued at an elevated level until it eventually made a significant decrease (see Fig 3D in [37]) presumably at the left saddle node seen in Fig 2D (right). Interestingly, when plotting the bistable switch of phosphopaxillin P* with respect to *B* (S1A Fig), we obtained a Michaelis-Menten-like profile with the two stable branches appearing close to each other. This indicated that, unlike *ρ* and R*, the level of P* changed markedly within the induced and uninduced states and that bistability was less pronounced for P* (making it less likely to be detected experimentally using fluorescent phosphopaxillin).

#### Effects of paxillin dephosphorylation rate *δ*_P_

The stability of the system could be also altered by varying the paxillin dephosphorylation rate, *δ*_*P*_. Because a change in *δ*_*P*_ is equivalent to an opposite change in *B*, we would expect the effect of *δ*_*P*_ to be simply the inverse of that of *B*. Since bistability existed for values of *δ*_*P*_ in the range of (0.0005, 0.0055), decreasing *δ*_*P*_ to below the lower bound of this interval would allow switching from the uninduced state to the induced state and increasing *δ*_*P*_ to above the upper bound would reverse the switch (S1B Fig). We observed evidence of this behaviour experimentally using a FRET-based Rac1 biosensor (which assesses the level of active Rac within the cell) and okadaic acid (OA), an inhibitor of paxillin phosphatase PP2A (S2A Fig) [65]. The cells expressed heterogeneous levels of active Rac and active Rho at the start of the experiment (i.e., different initial levels of active Rac and active Rho). Since adding OA inhibits paxillin dephosphorylation, incubation with the drug represents a decrease in *δ*_*P*_ in the model, while washout of the drug represents a recovery of *δ*_*P*_ to normal levels.

In general, the Rac activity across the cells increased following 20 mins of OA treatment (S2A Fig). Comparing the shape of the distribution of the average Rac activity (FRET ratios) per cell under the different conditions showed a rightward shift of the distribution to higher Rac activity following treatment with 20 nM of OA (S2B Fig, middle panel) compared to the distribution observed in control cells before adding OA (left panel). These results suggest that, as a population, cells exhibit increased Rac activity when paxillin dephosphorylation is inhibited. In fact, without OA, only 58% of cells exhibited high Rac activity (likely due to the presence of bistability between the induced and uninduced states), but 85% of cells exhibited high Rac activity after treatment with OA. After an extended 20 min washout period, there was partial recovery to baseline Rac activity as seen by a modest leftward shift of the distribution (right panel), with 70% of cells still exhibiting high Rac activity. The limited recovery could be due to cellular heterogeneity, incomplete washout, long term reactions to OA (>20 min), Rac activation pathways that are independent of paxillin, or many cells (but not all) crossing the left saddle node bifurcation of Fig 2D which, as suggested earlier, is very close to *B* = 0 s^-1^ (i.e, the vertical axis), limiting complete recovery.

### The GIT-PIX-PAK complex is needed to observe the effects of paxillin S273 phosphorylation

Downstream of paxillin phosphorylation, the binding between Pax_p_ and an intact GIT-PIX-PAK complex were shown to be required for the effects of paxillin S273 phosphorylation to be detected [25]. Disruption of the Pax_p_-GIT, GIT-PIX, or PIX-PAK interactions in D mutant cells caused a decrease in protrusiveness and a decline in adhesion assembly and disassembly rates. These phenotypes would be expected to arise from decreasing Rac activity, an outcome that was confirmed by the model described by Eqs (7)–(11) (see Methods Section). Indeed, after eliminating the Pax_p_-GIT, GIT-PIX, or PIX-PAK interactions in the model, through setting their respective association constants *k*_*C*_ = 0 s^-1^, *k*_*G*_ = 0 s^-1^, or *k*_*X*_ = 0 s^-1^, we found that the induced state cannot be reached, even after setting initial level of phosphopaxillin $P_{0}^{*}=1$.

GIT-PIX binding could be disrupted experimentally in two different ways: by either generating a GIT binding-deficient mutant of PIX (labeled PIX-ΔGBD) or by generating a PIX binding-deficient mutant of GIT (labeled GIT-ΔSHD). Transfecting CHO-K1 cells with the PIX-ΔGBD mutant did not cause any changes in the general behaviour of the cells (S3 Fig), as their migration speed (A), average adhesion size (B) and adhesion assembly/disassembly rates (C and D, respectively) remained effectively unchanged when compared to those of cells expressing wild type PIX. These results were consistent with previous observations showing that adhesion disassembly rate remained unchanged when D mutant cells were transfected with PIX-ΔGBD, in contrast to cells transfected with GIT-ΔSHD which exhibited a significantly slower adhesion disassembly rate [25]. The observed effect of the GIT-ΔSHD mutant was supported by the model, which showed that the induced state became unattainable when *k*_*G*_ = 0 even if $P_{0}^{*}=1$. The apparent discrepancy between the effects of the PIX-ΔGBD and GIT-ΔSHD mutants is likely due to the fact that CHO-K1 cells, transfected with PIX-ΔGBD, also express endogenous PIX, making these cells overexpress this protein (unlike the GIT-ΔSHD transfected cells). That is, the overexpression of PIX could lead to high PIX activity across the cell, and as a result cause more dynamic adhesions in spite of the lack of PIX binding to GIT.

**Fig 3:**
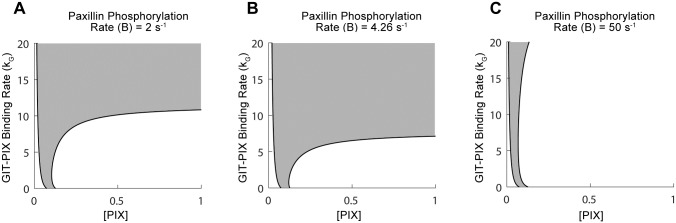
The effects of free PIX concentration ([*PIX*]) on bistability. Two-parameter bifurcation of the model with respect to the GIT-PIX binding rate, *k*_*G*_, and [*PIX*] demarcating the regime of bistability (gray) and monostability (white) associated with the uninduced (to the left of the bistable regime) and induced (to the right of the bistable regime) states. The regime of bistability shrinks as the value of maximum paxillin-phosphorylation rate *B* increases from 2 s^-1^ (A), to 4.26 s^-1^ (B) to 50 s^-1^ (C). The boundary of the bistable regime is defined by the two curves of saddle nodes.

To test this latter hypothesis, we plotted in Fig 3 the two-parameter bifurcation of the model, described by Eqs (7)–(11) (see Methods Section), with respect to GIT-PIX binding rate (*k*_*G*_) and PIX concentration ([*PIX*]), to trace the location of the two saddle nodes delimiting the bistable regime of Fig 2D, as both of these parameters are changed simultaneously. Our results revealed that the boundary of the bistable regime (gray area, bounded to the left by a very small monostable regime of uninduced states (white) and to the right by a large monostable regime of induced state (white)), are gradually dominated by the monostable regime of induced states, shrinking as the value of the maximum paxillin-phosphorylation rate increased from *B* = 2 s^-1^ (A) to its default value of *B* = 4.26 s^-1^ (B) and finally to *B* = 50 s^-1^ (C). In all cases, increasing [*PIX*] always mediated switching to the monostable induced state when *k*_*G*_ = 0, independent of the current state of the system (i.e., being induced or uninduced) and of the initial level of phosphopaxillin ($P_{0}^{*}$). This suggests that PIX overexpression induces Rac activation even if GIT-PIX binding is impaired, an outcome that is in agreement with the PIX-ΔGBD results of S3 Fig.

### PAK activation can also alter the stability of Rac and Rho

#### Effects of PAK activation rate α_R_

**Fig 4:**
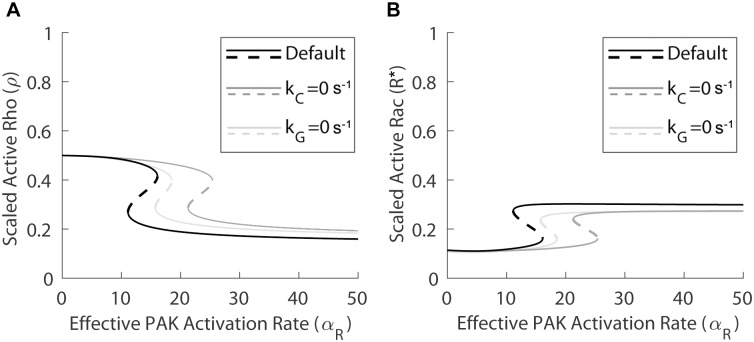
The effect of varying PAK-RacGTP binding rate (*α*_*R*_) depends on the Pax_p_-GIT and GIT-PIX interactions. Bifurcation diagrams of active Rho *ρ* (left) and active Rac R* (right) with respect to *α*_*R*_, showing the steady state levels of these two variables in the induced (elevated R*) and uninduced (elevated *ρ*) states; solid lines represent stable steady states, dashed lines represent saddle points. Under normal conditions (black curves), the system is bistable for *α*_*R*_ ∈ (11.1, 16.1). After setting *k*_*C*_ = 0 to eliminate the Pax_p_-GIT interaction (dark gray curves), the bistable regime shifts rightward to (21.3, 25.4), making it more difficult (easier) to reach the induced (uninduced) state. After setting *k*_*G*_ = 0 to eliminate the GIT-PIX interaction (light gray curves), the bistable regime also shifts rightward to (15.7, 18.5).

Since constitutively active and kinase dead variants of PAK have been shown to mimic the effects of the paxillin D and A mutants, respectively, and since the inhibition of PAK activation by IPA-3 reduces migration speed and adhesion assembly/disassembly rates, we investigated whether the effects of changing *α*_*R*_, the effective PAK activation rate, would also exhibit similar effects to those seen when changing the maximum paxillin-phosphorylation rate *B* in the model (Eqs (7)–(11) in Methods Section). The bifurcation diagrams of active Rho *ρ* (A) and active Rac R* (B) with respect to *α*_*R*_ in Fig 4 (black solid lines representing stable induced and uninduced states separated by a black dashed line of saddle points) showed that bistability was present when *α*_*R*_ ∈ (11.1, 16.1), and monostable regimes of uninduced and induced states existed for values of *α*_*R*_ below and above this range, respectively. These results imply that the system is sensitive to both increases and decreases in *α*_*R*_, which is in agreement with previous experimental observations [25,37]. Compared to the case with the maximum paxillin-phosphorylation rate *B* (see Fig 2D), the monostable regime of uninduced states was larger, likely due to the fact that PAK acts both upstream and downstream of paxillin [25].

#### PIX-PAK and PIX-PAK-RacGTP protein complexes are crucial for inducing the system

As with paxillin S273 phosphorylation/dephosphorylation, if the PIX-PAK interaction was disrupted (by making *k*_*X*_ = 0), bistability unsurprisingly disappeared into a monostable line of uninduced states, indicating that the effect of PAK activation/inactivation was eliminated. On the other hand, if the Pax_p_-GIT or GIT-PIX interactions were eliminated, by setting *k*_*C*_ = 0 s^-1^ or *k*_*G*_ = 0 s^-1^ respectively, then the branch of induced states persisted, but the regime of bistability shifted rightward, making it harder for the system to reach the monostable regime of induced states. This is evident from the bifurcation diagrams in Fig 4 (dark gray and light gray lines, designed in a similar fashion to black lines), showing that bistability was present for *α*_*R*_ ∈ (21.3, 25.4) and *α*_*R*_ ∈ (15.7, 18.5), respectively. The results of these simulations suggest that increasing the activation rate of PAK can shift the cells to the induced state without altering paxillin S273 phosphorylation and binding of Pax_p_ to the GIT-PIX-PAK complex. The reason for observing this effect is that the increase in Rac activity occurs through an increase in the levels of the PIX-PAK-RacGTP and PAK-RacGTP complexes. These complexes, which form independently of the Pax_p_-GIT and GIT-PIX complexes, increase the level of Rac activity by making the total level of PIX accumulate (through increased formation of the PIX-PAK-RacGTP complex) and by keeping Rac in the active state through binding to PAK (as determined by the value of *α*_*R*_).

Since disrupting the PIX-PAK interaction, but not the Pax_p_-GIT or GIT-PIX interactions, eliminated the branch of induced states, we hypothesized that the preservation of induced states in the latter two cases would be attributed primarily to PIX-PAK-RacGTP formation rather than that of PAK-RacGTP. Indeed, after omitting the formation of the PIX-PAK-RacGTP complex from the system (by excluding reactions 9–10 in S1 Table), the induced state disappeared upon the removal of the Pax_p_-GIT and GIT-PIX interactions (by setting *k*_*C*_ = 0 s^-1^ and *k*_*G*_ = 0 s^-1^, respectively). In other words, without the PIX-PAK-RacGTP complex, we were not able to recover the ability of the model to reach the induced state when the events occurring downstream of paxillin phosphorylation were disrupted.

#### Effects of total PAK-to-total Rac ratio *γ*

**Fig 5:**
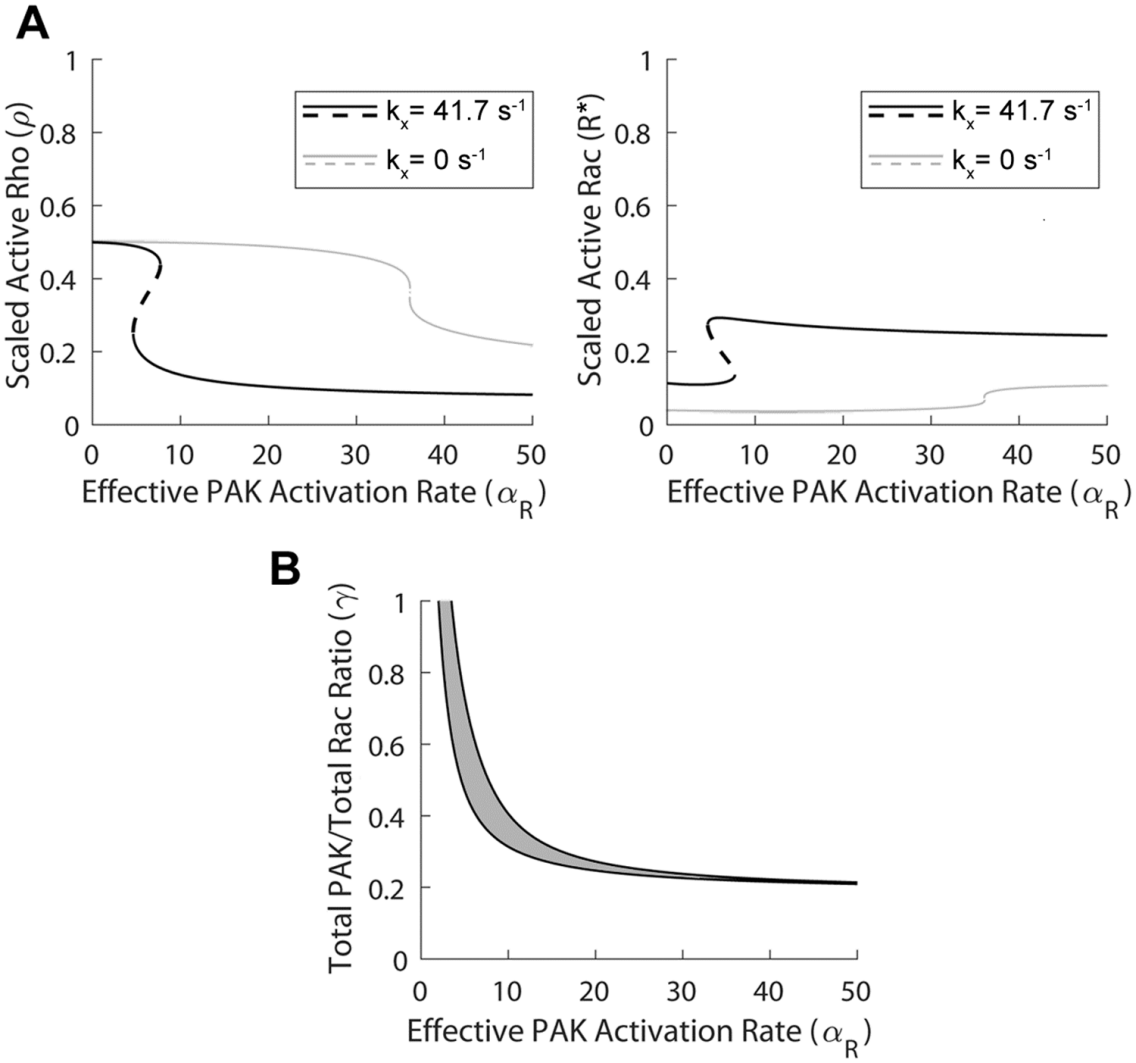
Dependence of PAK activation rate (*α*_*R*_) on [*PAK*_*tot*_]-to-[*Rac*_*tot*_] ratio (*γ*) and PIX-PAK binding rate (*k*_*X*_). (A) Bifurcation diagrams of active Rho *ρ* (left) and active Rac R* (right) with respect to *α*_*R*_ when *γ* = 0.5, showing the steady state levels of these two variables in the induced (elevated R*) and uninduced (elevated *ρ*) states; solid lines represent stable steady states, dashed lines represent saddle points. Although the induced state is conserved for both *k*_*X*_ = 41.7 s^-1^ (default value) (black lines) and *k*_*X*_ = 0 s^-1^ (gray lines), bistability regime in the latter case is much smaller and it exhibits a significant rightward shift. (B) Two-parameter bifurcation of the model with respect to *α*_*R*_ and *γ*, demarcating the regimes of bistability (gray) and monostability (white) associated with the uninduced (to the left of the bistable regime) and induced (to the right of the bistable regime) states. The boundary of the bistable regime is defined by the two curves of saddle nodes. Notice that when *γ* is large, PIX-PAK binding plays a smaller role in causing the induced state (compared to when the value of *γ* is smaller).

Since PIX-PAK binding and the formation of the PIX-PAK-RacGTP complex were necessary for the induced state to be attainable, the model showed that a large part of the increase in Rac activation associated with an increase in *α*_*R*_ was attributable to an increase in the total PIX level. It was possible, however, to diminish this contribution of PIX by selecting a high value for *γ* = [*PAK*_*tot*_]/[*Rac*_*tot*_]. In particular, setting *γ* = 0.5, as in Fig 5A, still produced bistability of induced and uninduced states for both active Rho *ρ* (left) and active Rac R* (right), even when PIX-PAK binding was disrupted by setting *k*_*X*_ = 0 s^-1^, but that was not the case for *γ* = 0.3 (results not shown). Furthermore, when the events downstream of paxillin phosphorylation were disrupted, the recovery of the induced state was still achievable by increasing PAK activation rate *α*_*R*_ even when the formation of the PIX-PAK-RacGTP complex was excluded from the set of reactions in S1 Table. It should be noted, however, that for all of the aforementioned cases, the bistable regime was very small and shifted towards the right, suggesting that it is more difficult to reach the induced state, and that the changes in the levels of active Rac and Rho become more gradual. The two-parameter bifurcation of the model with respect to *γ* and *α*_*R*_ (Fig 5B), which traces the location of the two saddle nodes of Fig 5A as both of these parameters are changed simultaneously, showed how the width of the *α*_*R*_-dependent bistable regime (gray area, flanked by monostable regimes of uninduced and induced states to the left and right, respectively) varied with *γ*. As the value of *γ* increased, the regime of bistability widened and shifted to the left, making it easier to reach the induced state through changes in *α*_*R*_. Note, however, that for high values of *α*_*R*_, the difference between the induced and uninduced states within the bistability regime was negligible and that below a threshold of *γ* ≈ 0.2, the system was always monostable (consisting of only the uninduced state) regardless of the value of *α*_*R*_.

### Spatial differences in paxillin phosphorylation can aid in establishing gradients implicated in determining cell polarity and directionality

**Fig 6:**
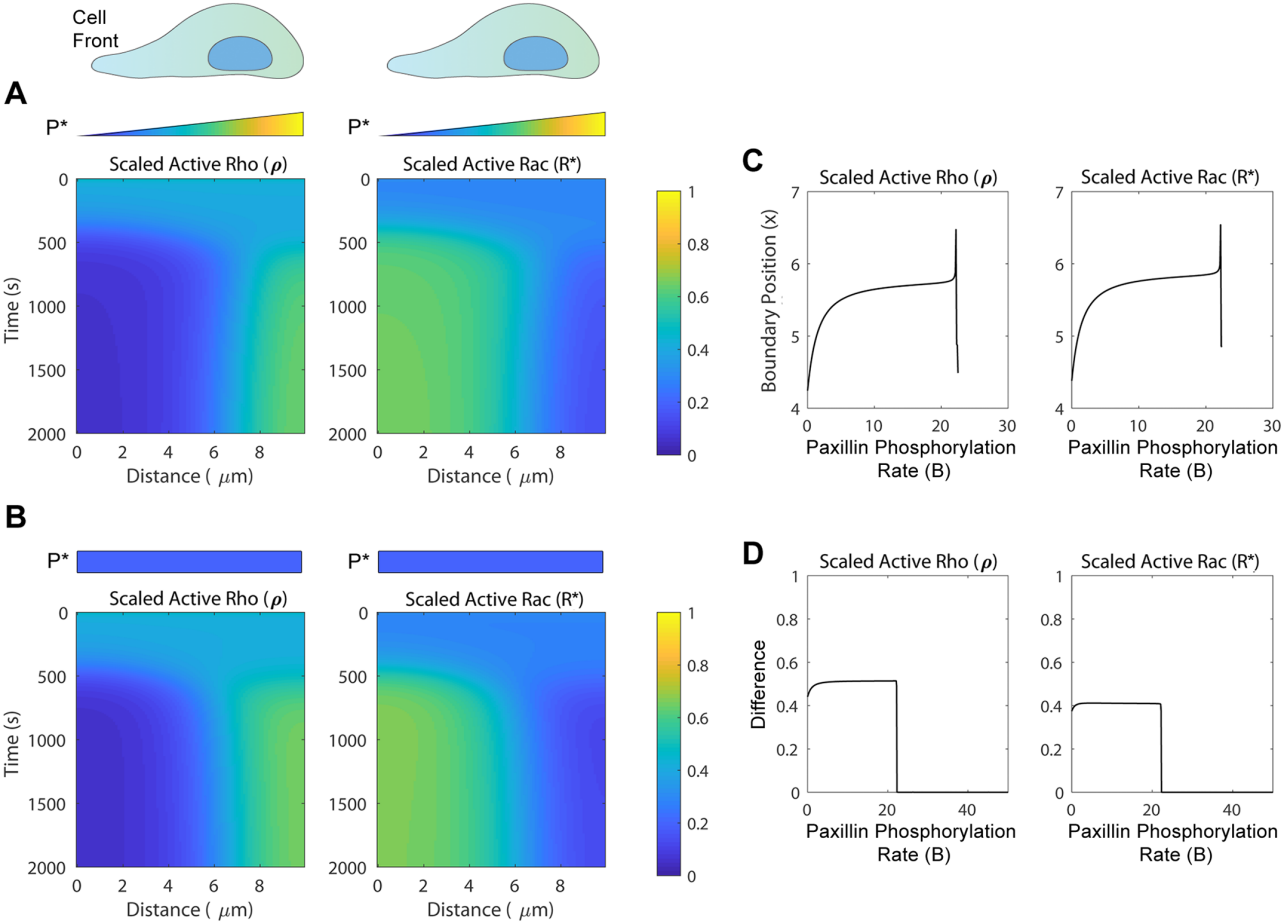
Heat-maps showing polarization in the spatiotemporal dynamics of active Rho (*ρ*) and active Rac (R*). (A) Simulation of the spatiotemporal model, starting with uniform initial levels of active Rho *ρ* and active Rac R*, and a sigmoidally decreasing gradient in initial level of phosphopaxillin P* with respect to *x* (i.e., high near *x* = 0 μm and low near *x* = *L* μm). (B) Simulation of the spatiotemporal model, with uniform initial levels of *ρ*, R* and P*, but with a sigmoidally decreasing maximum paxillin-phosphorylation rate *B* with respect to *x* (i.e., high near *x* = 0 μm and decays linearly to 0 as *x* increases). In both (A) and (B), active Rho *ρ* (left) and active Rac R* (right) have two distinct regions of activity which persist, indicating a polarization-like effect with a front (near *x* = 0 μm) and back (near *x* = *L* μm). (C) The *B*-dependent location of the boundary between the two polarized regions of Rac and Rho activity seen in (A). The boundary is defined as the position in space where the level of active Rho *ρ* (left), or active Rac R* (right) is halfway between their maximum and minimum levels. This boundary shifts to the right (i.e. towards the back of the “cell”) as *B* is increased until approximately *B* = 22.2 s^-1^, where it disappears due to the loss of active Rac/Rho gradient. (D) The *B*-dependent difference in the levels of active Rho *ρ* (left) and active Rac R* (right) obtained in the two polarized regions of panel (A). Notice how the difference disappears at *B* = 22.2 s^-1^, indicating loss of polarity.

Given that paxillin S273 phosphorylation/dephosphorylation rates appeared to play a key role in determining the steady state levels of active Rac and Rho, we asked next whether varying the level of phosphopaxillin in space could cause spatial gradients of Rac and Rho activity to form. To do so, we simulated our full spatiotemporal model of Rac/Rho activation and paxillin phosphorylation, given by Eqs (1)–(6) (see Methods Section), starting from spatially uniform initial levels of active Rac R*, and active Rho *ρ*, and a gradient of phosphopaxillin P*, that decreased sigmoidally from the cell front to the rear (see the heat-maps of Fig 6A). These initial conditions led to polarization-like effects in active Rac R* (right) and active Rho *ρ* (left), generating a region of high Rac/low Rho activity near the front of the cell at *x* = 0 μm, and a second region of low Rac/high Rho activity near the cell rear at *x* = *L* μm. This polarization-like effect closely resembled those previously seen and characterized in computational models of Rac-Rho cycling, in which decelerating (post transient) stationary waves were formed in a phenomenon termed wave-pinning [13,14,66]. Defining the boundary between the two regions of high Rac/low Rho and low Rac/high Rho as the value of *x* where the levels of the active forms of both GTPases were halfway between their maximum and minimum levels, we saw that as the maximum paxillin-phosphorylation rate *B* increased from *B* = 0 s^-1^, the position of the boundary initially increased sharply from *x* ≈ 4.25 μm, followed by a plateauing phase and then by a second sharp increase towards *x* ≈ 6.5 μm near *B* ≈ 22 s^-1^ (see Fig 6C). In other words, the boundary between the two polarized regions of Fig 6A gradually shifted to the back of the cell as *B* increased. Beyond *B* ≈ 22 s^-1^, the difference between the levels of these two GTPases in the two regions of polarization dropped significantly and became very small and visually indistinguishable from each other (Fig 6D). Effectively, this suggests that when *B* > 22 s^-1^, there is a relatively uniform level of both R* and *ρ* throughout and a loss of polarization in Rac and Rho activities.

Since spatial differences in the initial levels of P* were indeed capable of inducing polarization-like effects, we next examined whether a spatial difference in the maximum paxillin-phosphorylation rate, *B*, could generate a similar gradient of paxillin S273 phosphorylation and, in turn, have similar effects to those seen in Fig 6A. By starting with spatially uniform levels of *ρ*, R*, and P*, and values of *B* which decreased sigmoidally from the cell front to rear, the heat-maps of Fig 6B revealed that it was indeed possible for the model to generate distinct regions of Rac R* (right) and Rho *ρ* (left) activities that remained stable over time. It is important to point out that since the system was very sensitive to increases in *B*, we found that this polarization could only occur when the spatially uniform initial level of P* was very low (at most P* ≈ 0.1). If the initial level of P* was too high, the system became uniformly induced (an expected outcome in view of Fig 2A).

## Discussion

Paxillin phosphorylation at S273 has been shown to play a crucial role in determining cell movement. This was shown in CHO-K1 cells, expressing two different paxillin-S273 mutants (A and D), that exhibited distinct phenotypes in terms of adhesion dynamics and cellular motility (slow versus fast, respectively). Such dichotomy in the dynamics of adhesions along with paxillin suggests the presence of bistability, a common feature in many biological systems. To investigate this very feature and the underlying dynamics of this system, we developed a molecularly explicit mathematical model to examine how paxillin phosphorylation and its binding to the GIT-PIX-PAK complex affect Rac activation. The model was based on previous models of crosstalk between GTPases that exhibited bistability [13,14]. In the presence of several positive and negative feedback loops that directly and indirectly affect the Rac-Rho subsystem, the model preserved its bistable switch. Indeed, for the parameter values detailed in Table 1, we found that the model resulted in two stable steady states, one corresponding to the induced state (high Rac/low Rho activity) and another corresponding to the uninduced state (low Rac/high Rho activity). The bistable switch exhibited very pronounced difference between the induced and uninduced states in active Rac and Rho, but not in phosphopaxillin, and displayed features that were more in line with observed experimental data. In this study, we analyzed the steady state properties of the model and showed how perturbing various components of it affects outcomes. That allowed us to compare different model variations and identify the role of paxillin, PIX, PAK, *γ* = [*PAK*_*tot*_]/[*Rac*_*tot*_] and other molecular intermediates (such as GIT-PIX, GIT-PIX-PAK, PIX-PAK and PIX-PAK-RacGTP complexes) in defining dynamics, particularly bistability.

The bistable switch produced by the model was shown to be not only dependent on the paxillin S273 maximum phosphorylation rate, but also on its dephosphorylation rate. The bifurcation diagrams of active Rho and Rac with respect to the phosphorylation (dephosphorylation) rate exhibited a regime of bistability flanked to the left (right) and right (left) by the monostable regimes of uninduced and induced states, respectively. The existence of this bistable regime (bounded by two saddle nodes) produced a memory effect, where the final state of the system (whether induced or uninduced), obtained by varying one of these two parameters, was not necessarily the same as that obtained at default value. Furthermore, contrary to the bistable switch obtained by the model presented in [37], the levels of active Rac and Rho in the induced and uninduced states varied marginally within each state in the bistable switch, allowing for the level of active Rac (along with active Rho) to show a plateauing profile in the induced and uninduced states similar to that seen when inhibiting PAK activation with different concentrations of IPA-3 [37]. Although the two models had several common features, they differed in three main aspects: their parameter values, their detailed molecular nature, and their description of how RhoGTP affects Rac activity. For the latter, the model presented here assumed (as in [8,44,45]) that the active forms of Rac and Rho mutually inhibit each other by downregulating each other’s GEFs, but in [37], active Rho was assumed to activate Rac inactivation through GAP. Replacing Rho-dependent inhibition of Rac-GEF by Rho-dependent activation of Rac-GAP in the model could produce similar or related outcomes to those obtained here [14], but not necessarily within the same parameter regime identified as being physiologically-relevant (due to the dependence of several reactions of the model on the steady state expressions of other intermediates). Thus, it would be interesting to study how changing the effect of active Rho on Rac in the model alters/shift dynamics within the parameter space.

In agreement with experimental results, the effects of PAK activation/inactivation on the dynamics of Rac and Rho qualitatively mimicked those of paxillin S273 phosphorylation/dephosphorylation. We also discovered that, unlike with the maximum paxillin-phosphorylation rate, it was possible to recover the induced state by increasing the PAK activation rate even if the Pax_p_-GIT or GIT-PIX interactions were disrupted. The formation of the PIX-PAK-RacGTP complex was shown to be necessary for this recovery, highlighting the importance of this complex in ways not previously identified experimentally. Interestingly, if the value of total PAK-to-total Rac ratio (i.e., *γ*) was increased from its default value of 0.3 to the unphysiological value of 0.5, we saw a more robust recovery to the induce state, even if the S273 paxillin signalling pathway was impaired. In fact, we found that, at higher *γ*, this recovery no longer required the existence of the PIX-PAK-RacGTP complex, although recovery was more difficult in the absence of this complex. The results obtained for both cases (i.e., *γ* = 0.3 and *γ* = 0.5), led to the idea that PAK could recover Rac activation even when there were deficiencies in either paxillin S273 phosphorylation or formation/binding of the GIT-PIX-PAK complex, achieved through hyper-activation of PAK. All these model predictions involving PIX-PAK-RacGTP and *γ* remain to be validated experimentally.

Simulation of the full spatiotemporal (reaction diffusion) model suggested that a spatial difference in the level of phosphopaxillin or in the rates of paxillin S273 phosphorylation/dephosphorylation could produce gradients in Rac and Rho activity across the cell. Based on this, we concluded that regulation of paxillin S273 phosphorylation may aid in establishing polarity gradients and cell directionality through Rac. The polarization-like effects we observed were similar to those seen in minimal models of Rac-Rho interplay [38]. Using these minimal models, Holmes et al. demonstrated the existence of parameter regimes corresponding to cell spreading, rounding, and polarization. It is likely that these regimes also exist for the model presented here, and is a possible avenue of further pursuit. Interestingly, experimental evidence has previously shown that phosphopaxillin appeared in adhesions near the leading edge of the cell [25], accompanied by Rac activation prior to and during lamellipodial protrusion [9]. These results, along with the findings of the current study, suggested the possibility of a relationship between paxillin S273 phosphorylation and lamellipodial Rac activity that may lead to the propagation or maintenance of lamellipodial protrusions.

In addition to implications in establishing polarity gradients, phosphorylation of paxillin S273 may be related to certain cancerous phenotypes through deregulation of its phosphatase PP2A [4,67]. Inhibition and truncation of PP2A led to increased motility and metastasis [28,68], leading to the conclusion that certain cancerous phenotypes may be reversed by increasing PP2A activity to subsequently decrease paxillin phosphorylation and thus Rac activity. Fig 2D suggested, however, that while inhibiting and inducing paxillin dephosphorylation could increase and decrease the levels of Rac activity, respectively, switching from a pathological state to a physiological state may not be as intuitive as returning PP2A activity to normal physiological levels. If the range of normal dephosphorylation rates were within the bistable regime, for example, then the dephosphorylation rate would have to be increased beyond normal levels (past the right saddle node in Fig 2D) in order for cells to return back to the uninduced state.

In order to validate the predictions presented here, the activities of Rac and/or Rho (or signaling molecules downstream of Rac and Rho) must be measured while *B* and *α*_*R*_ are experimentally varied. One of the techniques used to achieve this is to measure the activities of FRET-based Rac and Rho biosensors while using chemical potentiators or inhibitors that activate or inhibit protein activities [41]. Here, we administered OA to decrease the paxillin dephosphorylation rate through the inhibition of PP2A. Although the cells had varying initial levels of active Rac, incubation of these cells with OA led to increased Rac activity, followed by partial recovery to normal levels after washout. We hypothesized that the absence of complete recovery to the normal level (after washout) was due to both the the presence of hysteresis and the close proximity of the left saddle node of Fig 2D to the vertical axis of that bifurcation diagram, limiting the recovery through this pathway. Experiments that detect the memory predicted in this system can also be done by measuring the activities of Rac and Rho at different concentrations of OA in a manner similar to what was done in [37] when measuring Rac and Rho activities in response to IPA-3. When the concentration of OA is increased from zero, Rac activity would be expected to increase as more cells enter the induced state. If, in the same experiment, the concentration of OA is gradually decreased, we would expect a decrease in the level of Rac activity. To demonstrate bistability, the EC_50_ (Rac)/IC_50_ (Rho) of the OA-dependent dose response curves obtained from the two experiments should be different.

Bistability in our model was verified by considering the assembly and disassembly rates of adhesion in wild type cells as well as in paxillin S273 A and D mutant cells. These rates were taken to be reflective of Rac and Rho activities, as faster (slower) assembly/disassembly rates were associated with high (low) Rac/low (high) Rho activities. Our results revealed that when plotting these rates in terms of adhesion sizes, wild type cells exhibited bimodality in their assembly/disassembly rates. Smaller adhesions were more dynamic while larger adhesions had slower assembly and disassembly rates. Interestingly, these rates were unimodal for the A and D mutants; the former exhibited only slow rates and the latter exhibited only high rates independent of adhesion size. We suggested that differences in initial level of phosphopaxillin (i.e., all molecular species containing Pax_p_) was responsible for generating the two subpopulations of adhesions in cells expressing wild-type paxillin, while differences in the maximum phosphorylation rate of paxillin were responsible for the appearance of only one subpopulation for both the A and D mutants.

Shifts and changes in the bistable switch obtained by the model when introducing certain binding deficiencies similar to those tested here (e.g., disrupting Pax_p_-GIT, GIT-PIX, or PIX-PAK interactions), could be qualitatively validated experimentally by checking for frequencies of adhesions with fast vs. slow assembly and disassembly rates when such deficiencies were introduced. This was done with the GIT-PIX intermediate (but not for PIX-PAK intermediate), in which CHO-K1 cells were transfected with the GIT binding-deficient mutant of PIX. These cells did not exhibit any changes in their adhesion assembly/disassembly, in contrast to those transfected with the PIX binding-deficient mutant of GIT [25]. Using the model, we showed that the overexpression of PIX (a known Rac-GEF) in the former case caused these cells to become induced regardless of the state of the system prior to transfection and of the initial level of phosphopaxillin ($P_{0}^{*}$).

This study clearly demonstrates the value of combined molecular modeling and experimental work. The model can now be used to guide future experiments, which in turn can help refine the model. In our future efforts, we aim to extend these approaches to further explore the molecular details of other intermediates regulating cell migration, and to develop phenomenological, but physiologically relevant, low dimensional model (based on the interactions between the three key variables: active Rho, active Rac and phosphopaxillin, incorporated into the model presented here) to study analytically cellular polarization and the phenomenon of wave-pinning [66].

## Supporting information

S1 Text Model derivation and parameter estimation. (PDF)

S1 Fig Effects of paxillin phosphorylation and dephosphorylation on bistability. (A) Bifurcation diagram of phosphopaxillin P* with respect to the maximum paxillin-phosphorylation rate *B*, showing the steady state levels of P* in the induced (elevated R*) and uninduced (elevated *ρ*) states; solid lines represent stable steady states, dashed lines represent saddle points. Notice the sigmoidal profile of the bifurcation diagram and the small difference between the levels of P* in the induced and uninduced states (as compared to the larger difference between the levels of *ρ* and R* in the two states). (B) Bifurcation diagrams of active Rho *ρ* (left), and active Rac R* (right panel) with respect to the paxillin dephosphorylation rate *δ*_*P*_, showing the steady state levels of these two variables in the induced (elevated R*) and uninduced (elevated *ρ*) states; solid lines represent stable steady states; dashed lines represent saddle points.
(TIF)

S2 Fig Distributions of mean cellular Rac activity with administration of okadaic acid. (A) Rac activity in CHO-K1 cells, quantified as a mean FRET ratio of the Rac1-2G biosensor across the entire cell, showing the variations in Rac activity ranging from low (left) to high (right); color bar, indicating magnitude of FRET ratio, shown on the right. The scale bar for both images is 5 μm. (B) The distributions of mean FRET ratios across multiple CHO-K1 cells (*n* = 20 − 31) with no treatment with okadaic acid (left), after 20 min of 20 nM treatment with okadaic acid (middle), and after 20 min of washout (right). The distribution shifts rightward with administration of okadaic acid, and the percentage of cells with high FRET ratios (>1, i.e. to the right of red line) increases from ~58% to ~85%, indicating an overall increase in Rac activity after inhibition of the paxillin dephosphorylation rate *δ*_*P*_. After washout, the distribution shifts partially back to the left, with ~70% still exhibiting high FRET ratio.
(TIF)

S3 Fig The effects of the GIT-PIX binding rate (*k*_*G*_) on cellular migration and adhesion dynamics. Transfecting CHO-K1 cells with GIT binding-deficient mutant of PIX (PIX-ΔGBD) does not alter migration velocity (A), average adhesion size (B) or adhesion assembly /disassembly rates (C and D, respectively) of these cells when compared to cells expressing wild type (WT) PIX.
(TIF)

S4 Fig Data fitting and parameter estimations. (A) Estimation of inactivation rates of Rho and Rac. (Left) Temporal profiles of Rho inactivation when incubated with RhoGAP alone (black dots) or with both RhoGAP and GST-C21 (grey squares). (Middle) Temporal profile of Rho self-inactivation (black dots). (Right) Temporal profile of Rac self-inactivation (black dots). Data points were digitized and fitted to mono-exponentially decaying functions (dashed lines) to calculate inactivation rates of Rho (under different conditions) and Rac. (B) Two-parameter bifurcation of the model with respect to *L*_*ρ*_ and *I* (left panel) and *I*_*K*_ and *I*_*R*_ (right panel) used to provide upper bounds for *L*_*ρ*_, *I*_*R*_, and *I*_*K*_. The bistable regime (gray) lies between the two curves of saddle nodes, and the monostable regimes of induced and uninduced states lie above and below the bistable regime, respectively. (C) Estimation of *B*, *L*_*K*_, and *δ*_*P*_. (Left) Temporal profile of paxillin phosphorylation following treatment with 100 μM of FAK activator carbachol (black dots). Using the steady state fraction of phosphorylated paxillin, the initial phosphorylation rate, as well as Assumptions (1)-(4), we estimated the paxillin dephosphorylation rate *δ*_*P*_. (Middle) Dose response curve of paxillin phosphorylation when treated with varying concentrations of carbachol (black dots). (Right) Paxillin phosphorylation with respect to FAK activation (black dots) determined by combining the data in Middle panel with Western blot quantification of FAK response to carbachol treatment. The data points in the Right panel were fitted to Eq. S8 (dashed line), using a non-linear least squares fitting, to estimate the parameters *B* and *L*_*K*_.
(TIF)

S1 Table Complete list of reaction schemes between Rac, Rho, paxillin, and the GIT-PIX-PAK complex. Agents associated with the transition arrows (e.g., *RhoGEF* and *RhoGAP* in reaction 1) are catalysts for these reactions.
(PDF)

S2 Table Western blot quantifications to estimate the relative activation of FAK in response to carbachol administration. (PDF)
