# Supplementary material for: Paxillin phosphorylation at serine 273 and its effects on Rac, Rho and adhesion dynamics
Source: PLoS Comput Biol. 2018 Jul 5;14(7):e1006303. doi: 10.1371/journal.pcbi.1006303 (PMC6053249; doi:10.1371/journal.pcbi.1006303)
Supplement: S1 Text — (PDF) [file pcbi.1006303.s001.pdf]

S1 TEXT - Model Derivation and Parameter Estimation

Model Derivation

In addition to the equations defined for Rac, Rho, and paxillin, we have the reactions of the intermediate species (Reactions 3, 5-12 in S1 Table) which, in our model, are set to steady state. The steady state concentrations of these intermediates (derived here), will be used in the expressions for phosphorylated paxillin-dependent Rac activation rate (I_K^*) and the ratio of $[PAK]$-to-$[PAK_{tot}]$ (K_i^*). From S1 Table, the equations governing the dynamics of these intermediate species are given by

$$\frac{d[PAK]}{dt} = k_{PAK}^{-}[PAK\text{-}RacGTP] - k_{PAK}^{+}[PAK][RacGTP] + k_X^{-}[GIT\text{-}PIX\text{-}PAK] - k_X^{+}[GIT\text{-}PIX][PAK] + k_X^{-}[PIX\text{-}PAK] - k_X^{+}[PIX][PAK]$$

$$\frac{d[PAK\text{-}RacGTP]}{dt} = -k_{PAK}^{-}[PAK\text{-}RacGTP] + k_{PAK}^{+}[PAK][RacGTP] + k_X^{-}[PIX\text{-}PAK\text{-}RacGTP] - k_X^{+}[PIX][PAK\text{-}RacGTP]$$

$$\frac{d[PAK\text{-}RacGTP]}{dt} = -k_{PAK}^{-}[PAK\text{-}RacGTP] + k_{PAK}^{+}[PAK][RacGTP]$$

$$\frac{d[GIT\text{-}PIX]}{dt} = k_G^{+}[GIT][PIX] - k_G^{-}[GIT\text{-}PIX] + k_X^{-}[GIT\text{-}PIX\text{-}PAK] - k_X^{+}[GIT\text{-}PIX][PAK]$$

$$\frac{d[PIX\text{-}PAK]}{dt} = k_X^{+}[PIX][PAK] - k_X^{-}[PIX\text{-}PAK] + k_G^{-}[GIT\text{-}PIX\text{-}PAK] - k_G^{+}[GIT][PIX\text{-}PAK] + k_{PAK}^{-}[PIX\text{-}PAK\text{-}RacGTP] - k_{PAK}^{+}[PIX\text{-}PAK][RacGTP]$$

$$\frac{d[PIX\text{-}PAK\text{-}RacGTP]}{dt} = k_X^{+}[PIX][PAK\text{-}RacGTP] - k_X^{-}[PIX\text{-}PAK\text{-}RacGTP] + k_{PAK}^{+}[PIX\text{-}PAK][RacGTP] - k_{PAK}^{-}[PIX\text{-}PAK\text{-}RacGTP]$$

$$\frac{d[GIT\text{-}PIX\text{-}PAK]}{dt} = k_X^{+}[GIT\text{-}PIX][PAK] - k_X^{-}[GIT\text{-}PIX\text{-}PAK] + k_G^{+}[GIT][PIX\text{-}PAK] - k_G^{-}[GIT\text{-}PIX\text{-}PAK] + k_C^{-}\left[Pax_p\text{-}GIT\text{-}PIX\text{-}PAK\right] - k_C^{+}[Pax_p][GIT\text{-}PIX\text{-}PAK]$$

$$\frac{d\left[Pax_p\text{-}GIT\text{-}PIX\text{-}PAK\text{-}RacGTP\right]}{dt} = -k_{PAK}^{-}\left[Pax_p\text{-}GIT\text{-}PIX\text{-}PAK\text{-}RacGTP\right] + k_{PAK}^{+}\left[Pax_p\text{-}GIT\text{-}PIX\text{-}PAK\right][RacGTP]$$

$$\frac{d[Pax_p\text{-}GIT\text{-}PIX\text{-}PAK]}{dt} = k_C^+[Pax_P][GIT\text{-}PIX\text{-}PAK] - k_C^-[Pax_p\text{-}GIT\text{-}PIX\text{-}PAK] + k_{PAK}^-[Pax_p\text{-}GIT\text{-}PIX\text{-}PAK\text{-}RacGTP] - k_{PAK}^+[Pax_p\text{-}GIT\text{-}PIX\text{-}PAK][RacGTP].$$

After setting the above equations to steady state using QSS approximation, the concentrations of each intermediate can be expressed as a function of the concentrations of RacGTP, phosphorylated paxillin, and monomeric GIT, PIX, and PAK. Their steady state expressions are given by

$$[PAK\text{-}RacGTP] = \frac{k_{PAK}^+}{k_{PAK}^-}[PAK][RacGTP]$$

$$[GIT\text{-}PIX] = \frac{k_G^+}{k_G^-}[GIT][PIX] = \text{constant}$$

$$[PIX\text{-}PAK] = \frac{k_X^+}{k_X^-}[PIX][PAK]$$

$$[PIX\text{-}PAK\text{-}RacGTP] = \frac{k_X^+ k_{PAK}^+}{k_X^- k_{PAK}^-}[PIX][PAK][RacGTP]$$

$$[Pax_P\text{-}GIT\text{-}PIX\text{-}PAK] = \frac{k_G^+ k_X^+ k_C^+}{k_G^- k_X^- k_C^-}[GIT][PIX][PAK][Pax_p]$$

$$[Pax_p\text{-}GIT\text{-}PIX\text{-}PAK\text{-}RacGTP] = \frac{k_G^+ k_X^+ k_C^+ k_{PAK}^+}{k_G^- k_X^- k_C^- k_{PAK}^-}[GIT][PIX][PAK][Pax_p][RacGTP].$$

The expression for the steady state concentration of monomeric PAK is derived from the total concentration of PAK (assumed to be constant), as follows

$$[PAK_{tot}] = [PAK] + [PIX\text{-}PAK] + [GIT\text{-}PIX\text{-}PAK] + [Pax_p\text{-}GIT\text{-}PIX\text{-}PAK] + [PAK\text{-}RacGTP] + [PIX\text{-}PAK\text{-}RacGTP] + [Pax_p\text{-}GIT\text{-}PIX\text{-}PAK\text{-}RacGTP].$$

Substituting the concentrations of the intermediate species for their steady state concentrations, we obtain

$$\frac{[PAK_{tot}]}{[PAK]} = k_X[PIX] + k_G k_X[GIT][PIX] + k_G k_X k_C[GIT][PIX][Pax_p] + \alpha k_X[PIX][RacGTP] + \alpha k_G k_X k_C[GIT][PIX][Pax_p][RacGTP] + 1 + \alpha[RacGTP], \quad \text{(S1)}$$

where $k_G = \frac{k_G^+}{k_G^-}$, $k_X = \frac{k_X^+}{k_X^-}$, $k_C = \frac{k_C^+}{k_C^-}$ and $\alpha = \frac{k_{PAK}^+}{k_{PAK}^-}$. Equation (S1) is equivalent to

$$[PAK] = \frac{[PAK_{tot}]}{\left(1+k_X[PIX]+k_G k_X k_C[GIT][PIX]\left[Pax_p\right]\right)(1+\alpha[RacGTP]) + k_G k_X[GIT][PIX]}.$$

From Eq. (S1), we define

$$K_i^* = \frac{[PAK]}{[PAK_{tot}]} = \frac{1}{\left(1+k_X[PIX]+k_G k_X k_C[GIT][PIX]\left[Pax_p\right]\right)(1+\alpha[RacGTP]) + k_G k_X[GIT][PIX]} .$$

To determine the phosphorylated paxillin-dependent Rac activation rate I_K^*, recall first that

$$I_K^* = I_K'\big([PIX\text{-}PAK] + [GIT\text{-}PIX\text{-}PAK] + \left[Pax_p\text{-}GIT\text{-}PIX\text{-}PAK\right] + [PIX\text{-}PAK\text{-}RacGTP] + \left[Pax_p\text{-}GIT\text{-}PIX\text{-}PAK\text{-}RacGTP\right]\big).$$

Substituting the concentrations of each intermediate species using their concentrations at steady state, we have

$$I_K^* = I_K'\Big([PAK]\big(k_X[PIX] + k_G k_X[GIT][PIX] + k_G k_X k_C[GIT][PIX]\left[Pax_p\right] + \alpha k_X[PIX][RacGTP] + \alpha k_G k_X k_C[GIT][PIX]\left[Pax_p\right][RacGTP]\big)\Big)$$

$$= I_K'\left([PAK]\left(\frac{[PAK_{tot}]}{[PAK]} - 1 - \alpha[RacGTP]\right)\right)$$

$$= I_K'[PAK_{tot}]\left(1 - \frac{[PAK]}{[PAK_{tot}]} - \frac{\alpha[PAK][RacGTP]}{[PAK_{tot}]}\right) .$$

Finally, substituting $I_K = I_K'[PAK_{tot}]$, $K_i = \frac{[PAK]}{[PAK_{tot}]}$, $R = \frac{[RacGTP]}{[Rac_{tot}]}$, and $\alpha_R = \alpha[Rac_{tot}]$, we obtain

$$I_K^* = I_K\big(1 - K_i^*(1+\alpha_R R)\big)$$

To determine the expression level of unphosphorylated paxillin, P_i, in Eq. (9), we first assume a constant total concentration of paxillin, which includes unphosphorylated and phosphorylated paxillin and any complexes which contain them. Given this assumption, the concentration of unphosphorylated paxillin is

$$[Pax] = [Pax_{tot}] - \left[Pax_p\right] - \left[Pax_p\text{-}GIT\text{-}PIX\text{-}PAK\right] - \left[Pax_p\text{-}GIT\text{-}PIX\text{-}PAK\text{-}RacGTP\right].$$

After substituting the steady state expressions for the appropriate intermediate complexes, we obtain

$$[Pax] = [Pax_{tot}] - [Pax_p]\big(1 + k_G k_X k_C [GIT][PIX][PAK](1 + \alpha[RacGTP])\big) \qquad \text{(S2)}$$

Finally, scaling Eq. (S2) to the total concentration of paxillin and substituting the scaled variables $P = \frac{[Pax_p]}{[Pax_{tot}]}, P_i = \frac{[Pax]}{[Pax_{tot}]}, K_i^* = \frac{[PAK]}{[PAK_{tot}]}$ and $R = \frac{[RacGTP]}{[Rac_{tot}]}$, give

$$P_i = 1 - P\big(1 + k_G k_X k_C [GIT][PIX][PAK_{tot}]K_i^*(1 + \alpha_R R)\big),$$

where $\alpha_R = \alpha[Rac_{tot}]$.

Parameter Estimation

The parameters of the system are estimated in part from values directly obtained from the literature and in part by inference and manipulation of parameter values that produce the observed experimental results. As in [1] and [2], we assume $n=4$ to establish a high degree of cooperativity in Rac inhibition, Rho inhibition, and paxillin phosphorylation. The remaining parameter estimations are detailed below.

Rho activation and inactivation

To measure the Rho inactivation rate δ_ρ, RhoGTP was incubated with Rho GAPs to determine how the level of RhoGTP, relative to its initial level, decayed over time [3]. This inactivation can be modeled as a decay occurring at a rate proportional to the concentration of RhoGTP, as determined by the equation

$$\frac{d\rho}{dt} = -\delta_\rho \rho.$$

Because RhoGTP is normalized by its initial value, then $\rho(0) = 1$, and the level of active Rho, as a function of time, is given by $\rho(t) = e^{-\delta_\rho t}$. The natural logarithm of ρ is therefore linear with respect to time with slope $-\delta_\rho$, given by

$$\ln \rho = -\delta_\rho t. \qquad \text{(S3)}$$

To estimate δ_ρ, the data for Rho inactivation is digitized using WebPlotDigitizer and the natural logarithm of Rho concentration is used to fit Eq. (S3) to data based on a linear least squares estimation [3] (S4A Fig, left). The magnitude of the slope of the fitted curve gives the estimate δ_ρ = 0.016 s^{-1}.

The activation rate I_ρ is estimated from Rho activation assays in suspended cells stimulated with lysophosphatidic acid, an activator of Rho [4]. In suspension, cells express very low levels of active Rac [5] and are therefore assumed to be negligible ($R^* \approx 0$). Rho activation is measured by its binding to GST-C21, a fragment of the protein Rhotekin, which binds specifically to GTP-bound Rho. In the presence of GST-C21, the Rho inactivation rate is reduced, thus causing Rho to inactivate at a reduced rate δ_ρ^* [3]. This means that the change in the concentration of active Rho in this system is determined by modifying Eq. (7) to

$$\frac{d\rho}{dt} = I_\rho(1-\rho) - \delta_\rho^* \rho. \qquad \text{(S4)}$$

If Eq. (S4) is set to steady state, the activation rate can be determined algebraically using the equation $I_\rho = \frac{\delta_\rho^* \rho_{SS}}{1-\rho_{SS}}$, where ρ_{SS} is the scaled level of active Rho at steady state. From experiments, we use the plateauing phase of the curve in the upper panel of Fig 5 obtained from [6] to estimate that $\rho_{SS} \approx 0.85$ in the presence of GST-C21. The reduced inactivation rate $\delta_\rho{}^*$ is then determined from Rho inactivation assays measured in the presence of GST-C21 [3]. Since RhoGTP was incubated in the absence of RhoGEFs, $\delta_\rho{}^*$ can be estimated using once again an exponential model of Rho inactivation. Fitting the model to the data in [3] gives $\delta_\rho{}^* = 0.0028$ s^{-1} and $I_\rho = 0.0159$ s^{-1}.

Finally, L_R, the level at which half-maximal Rho inhibition is reached, can be calculated by substituting the known parameter values and the steady state levels of active Rac and Rho. For this estimation, Rac and Rho activity must be measured from the same experiment in order to obtain a reliable comparison between the two variables within the same system. Thus, R_{SS}^* and ρ_{SS} are determined from quantification of Western blots from the same sample [7]. Based on the fold change in intensity between the blots for active and total protein, we find that $R_{SS}^* = 0.45$ and $\rho_{SS} = 0.19$. Using these concentrations and the derived parameter values obtained above, one can calculate the value of L_R at steady state using Eq. 7 in the Main Text, i.e.,

$$L_R = R_{SS}^* \sqrt[n]{\frac{\delta_\rho \rho_{SS}}{I_\rho(1-\rho_{SS}) - \delta_\rho \rho_{SS}}} = 0.336.$$

Rac activation and inactivation

As with Rho, the Rac inactivation rate, δ_R, is estimated from Rac inactivation assays as follows. Inactivation of both Rac and Rho can occur either intrinsically, i.e. by self-inactivation, or can occur through the activity of GTPase-specific GAPs. Using experimental data digitized from [3,8], the Rho self-inactivation rate (i.e. in the absence of Rho GAPs) was estimated to be 0.0024 s^{-1} (S4A Fig, middle), while the Rac self-inactivation rate was estimated to be 0.0012 s^{-1} (S4A Fig, right). From the previous section, the Rho inactivation rate in the presence of Rho GAPs was estimated to be 0.016 s^{-1}, a 6.5-fold increase from its self-inactivation rate. Assuming a similar fold change between the Rac inactivation rate in the absence and presence of Rac GAPs, the inactivation rate of Rac in the presence of GAPs, i.e. δ_R, is estimated to be $\delta_R = 0.0012 s^{-1} \times 6.5 = 0.009\, s^{-1}$. Inactivation rates were estimated following an exponential model of inactivation.

Exploring how the values of I_R, I_K, and L_R affect the dynamics of the system allows us to estimate the range of values that the three parameters can attain to produce bistability within the model. An appropriate bound is first determined for L_ρ after modifying Eq. (8) to

$$\frac{dR}{dt} = I\left(\frac{L_\rho^n}{L_\rho^n + \rho^n}\right)(1 - R^*) - \delta_R R,$$

where I is the activation rate of Rac (assumed to be constant). The existence of steady states is thus dependent on the values of I and L_ρ, and this dependence can be visualized in a two-parameter bifurcation diagram with respect to the two parameters. The L_ρ-I two-parameter bifurcation diagram in S4B Fig (left) shows a regime of bistability (gray area) between the two curves of saddle nodes, above and below of which lie the monostable regimes of induced and uninduced states, respectively. This two-parameter bifurcation diagram shows that the bistable regime is limited to the region where $L_\rho < 0.43$ (which represents an upper bound for L_ρ). A value of L_ρ is then chosen under the assumption that the activation rates of Rac and Rho are comparable to each other and with the constraint that L_ρ must be chosen in such a way that the bistable regime and both monostable regimes can be observed for reasonably small changes in I_R and I_K. We therefore choose $L_\rho = 0.34$, a value slightly below the upper bound and is equal to L_R.

To similarly define bounds for I_R and I_K, the two parameters are simultaneously varied in the two-parameter bifurcation of S4B Fig (right), which shows that the upper bounds for both parameters are relatively large at ~0.01 for both I_R and I_K. We choose intermediate values of $I_R = 0.003$ s^{-1} and $I_K = 0.009$ s^{-1}, allowing the value of I_K to be greater than that of I_R in order to highlight the augmentation of Rac activity arising from increased paxillin S273 phosphorylation.

The concentrations of GIT, PIX, and Pax$_{tot}$

The concentrations of GIT, PIX, and paxillin relative to that of Rac can be calculated from quantitative mass spectrometry, using intensity-based absolute quantification (iBAQ) [8]. Then, the total concentration of Rac1 can be estimated as in [1] and [2].

To determine relative protein concentrations using iBAQ, proteins were isolated from whole cell lysates, digested, and analyzed by mass spectrometry, resulting in a spectrum for each peptide of a given protein. The iBAQ number, which is an absolute protein copy number, was then calculated as the sum of all identified peptide intensities divided by the theoretical number of observable peptides. For each protein, three different digestion methods were used, giving rise to three different estimated concentrations. The iBAQ numbers of GIT, PIX, Paxillin, and Rac1 were on the ranges of 2.1×10^6–5.4×10^6, 2.6×10^6–4.3×10^6, 5.1×10^6–1.1×10^7, and 1.9×10^7–2.9×10^8 respectively.

The total concentration of Rac1 can be estimated as follows. The mass of Rac in COS1 fibroblasts is 82 ng/10^6 cells. Since the molecular weight of Rac is 21 kDa = 3.49×10^{-11} ng [1,2], the total number of Rac proteins in 10^6 cells is 2.35×10^{12} = 3.90×10^{-12} mol. Assuming spherical cells with diameters of 10 μm, each cell will have a volume of 523 μm^3, and 10^6 cells will have a total volume of 5.23×10^8 μm^3 = 5.23×10^{-10} m^3 = 5.23×10^{-7} L. Therefore, the total concentration of Rac is $\frac{3.90\times10^{-12}\text{mol}}{5.23\times10^{-7}\text{L}}$ =7.5 μM. Using this information, we can calculate the ranges of $[GIT]$, $[PIX]$, and $[Pax_{tot}]$ to be

$$[GIT]_{min} = \frac{[GIT]_{min}}{[Rac_{tot}]_{max}}[Rac_{tot}] = \frac{2.1\times10^6}{2.9\times10^8}\times 7.5\ \mu\text{M} = 0.054\ \mu\text{M}$$

$$[GIT]_{max} = \frac{[GIT]_{max}}{[Rac_{tot}]_{min}}[Rac_{tot}] = \frac{5.4 \times 10^6}{1.9 \times 10^7} \times 7.5\ \mu\text{M} = 2.1\ \mu\text{M}$$

$$[PIX]_{min} = \frac{[PIX]_{min}}{[Rac_{tot}]_{max}}[Rac_{tot}] = \frac{2.6 \times 10^6}{2.9 \times 10^8} \times 7.5\ \mu\text{M} = 0.069\ \mu\text{M}$$

$$[PIX]_{max} = \frac{[PIX]_{max}}{[Rac_{tot}]_{min}}[Rac_{tot}] = \frac{4.3 \times 10^6}{1.9 \times 10^7} \times 7.5\ \mu\text{M} = 1.7\ \mu\text{M}$$

$$[Pax_{tot}]_{min} = \frac{[Pax_{tot}]_{min}}{[Rac_{tot}]_{max}}[Rac_{tot}] = \frac{5.1 \times 10^6}{2.9 \times 10^8} \times 7.5\ \mu\text{M} = 0.13\ \mu\text{M}$$

$$[Pax_{tot}]_{max} = \frac{[Pax_{tot}]_{max}}{[Rac_{tot}]_{min}}[Rac_{tot}] = \frac{1.1 \times 10^7}{1.9 \times 10^7} \times 7.5\ \mu\text{M} = 4.5\ \mu\text{M}$$

From these ranges, we estimate the values of $[GIT]$, $[PIX]$ and $[Pax_{tot}]$ to be 0.11 µM, 0.069 µM and 2.3 µM, respectively. Note that while the concentrations of GIT and PIX are closer to their lower bounds, the concentration of paxillin is chosen to be relatively high in order for paxillin phosphorylation to have an effect on Rac/Rho activity.

The parameters $\mathbf{k_C}$, $\mathbf{k_G}$,$\mathbf{k_X}$, and $\boldsymbol{\alpha_R}$

The ratios of forward/backward rates are first estimated from the dissociation constants of GIT-PIX, PIX-PAK, and PAK-RacGTP available in the literature [9–12]. The values of k_G and k_X are left unaltered; however, the estimated value of $k_C = 0.1$ s^{-1} is too small (it made paxillin play no role in determining the stability of the system). To resolve this issue, we choose a higher value k_C given by 5 s^{-1}. This increase in the value of k_C is justified by the fact that Pax_p-GIT binding is augmented in the presence of PIX; since the dissociation constant (k_C^{-1}) of this binding was measured in the absence of PIX [12], its value is likely overestimated and its effective range of values is significantly lower in physiological conditions. Finally, since $\alpha_R = \alpha[Rac_{tot}] = \frac{k_{PAK}^+}{k_{PAK}^-}[Rac_{tot}]$, we conclude that $\alpha_R = 2.0 \times 7.5 = 15$.

Paxillin phosphorylation and dephosphorylation

The dephosphorylation rate of paxillin is estimated from assays of paxillin tyrosine phosphorylation, performed after stimulation with 100 µM carbachol, an activator of the tyrosine kinase FAK [13]. To estimate δ_P from these experiments, we assume the following:

1. FAK-mediated tyrosine phosphorylation on paxillin occurs at a rate similar to PAK-mediated serine phosphorylation, allowing the former estimate to be used for the PAK-mediated dephosphorylation rate.
2. The concentration of active FAK is comparable to that of active PAK, i.e. $\frac{[FAK(active)]}{[FAK(total)]} \approx \frac{[PAK\text{-}RacGTP^*]}{[PAK_{tot}]} = K$.
3. When stimulated with 100 μM carbachol, all FAK proteins are active, and therefore, by Assumption (2), all PAK molecules are active, i.e. $K \approx 1$.
4. The concentration of phosphorylated paxillin at the beginning of the assay is negligible, i.e. $P(0) \approx 0$.

Applying Assumption (3) gives the differential equation for phosphorylated paxillin in the presence of carbachol

$$\left.\frac{dP}{dt}\right|_{(K\approx 1)} = \frac{B}{L_K^n+1}(1-P) - \delta_P P.$$

Applying Assumption (4), we conclude that the initial phosphorylation rate of paxillin is

$$\left.\frac{dP}{dt}\right|_{(P\approx 0,K\approx 1)} = \frac{B}{L_K^n+1} \qquad \text{(S5)}$$

and the steady state concentration of paxillin in the presence of carbachol is

$$P_{(SS,K\approx 1)} = \frac{B}{B+\delta_P(L_K^n+1)}. \qquad \text{(S6)}$$

Using Eqs. (S5) and (S6) to solve for δ_P, we have $\delta_P = \frac{dP}{dt}_{(P\approx 0,K\approx 1)} \times \left(\frac{1}{P_{SS,K\approx 1}} - 1\right)$. By digitizing the data associated with the phosphorylation assay [13] (see S4C Fig, left), we find that $P_{(SS,K\approx 1)} = 0.83$ and $\left.\frac{dP}{dt}\right|_{(P\approx 0,K\approx 1)} = 0.0020$ s^{-1}. Therefore, the dephosphorylation rate of paxillin is estimated to be $\delta_P = 0.000413$ s^{-1}.

The parameters B and L_K are estimated by digitizing the dose response curves of carbachol-induced paxillin tyrosine phosphorylation [13] (see S4C Fig, middle). For each dose of carbachol, the relative concentrations of active FAK and tyrosine-phosphorylated paxillin are determined from quantified Western blot data [13]. To determine the level of tyrosine phosphorylated paxillin as a function of FAK activation, the anti-FAK blot is quantified using

ImageJ and normalized to the intensity of the lane corresponding to the highest dosage of carbachol treatment. The levels of FAK activation for each dosage are then taken to be the relative intensities of each corresponding lane (see S2 Table), and phosphorylated paxillin is quantified for each dosage of carbachol.

To estimate B and L_K from the dose response curves, we need first to set Eq. (9), appearing in the main text, to steady state

$$0 = B\left(\frac{K^n}{L_K^n + K^n}\right)\left(1 - P\left(1 + \frac{\bar{\beta}_P(1+\alpha_R R)}{\eta(\beta + \bar{\beta}_P P)}(1-K)\right)\right) - \delta_P. \qquad \text{(S7)}$$

Equation (S7) can be fit to the data by finding an explicit expression for P in terms of K and R and identifying a pair of parameter values B and L_K for which the theoretical (P,K) curve produces the best fit to the data. However, Eq. (S7) is highly nonlinear, and an explicit expression is difficult to derive. Moreover, R is unknown, since the level of active Rac was not measured in these experiments [13]. However, R, K, and P are all bounded between 0 and 1. This means that the expression $\frac{\bar{\beta}_P(1+\alpha_R R)}{\eta(\beta+\bar{\beta}_P P)}(1-K)$ is also bounded. Since $\frac{\bar{\beta}_P(1+\alpha_R R)}{\eta(\beta+\bar{\beta}_P P)}(1-K)$ increases monotonically with R and decreases monotonically with P and K, the upper bound of this expression occurs at $R = 1$, $P = 0$, and $K = 0$, i.e.,

$$\frac{\bar{\beta}_P(1+\alpha_R R)}{\eta(\beta+\bar{\beta}_P P)}(1-K) \le \frac{\bar{\beta}_P(1+\alpha_R)}{\eta\beta}, \qquad 0 \le R, K, P \le 1.$$

If this upper bound is negligibly small, then $\frac{\bar{\beta}_P(1+\alpha_R R)}{\eta(\beta+\bar{\beta}_P P)}(1-K)$ is also negligible for all values of R, P, and K within the range [0,1]. Given the previously estimated parameters, we find that the upper bound is

$$\frac{\bar{\beta}_P(1+\alpha_R)}{\eta\beta} = \frac{0.053(1+15)}{11.9 \times 5.69} = 0.01.$$

Since

$$1 + \frac{\bar{\beta}_P(1+\alpha_R R)}{\eta(\beta+\bar{\beta}_P P)}(1-K) \le 1 + \frac{\bar{\beta}_P(1+\alpha_R)}{\eta\beta} = 1.01 \approx 1,$$

we conclude that the contribution of the expression $\frac{\bar{\beta}_P(1+\alpha_R R)}{\eta(\beta+\bar{\beta}_P P)}(1-K)$ to Eq. (S7) is small, and that the assumption $\frac{\bar{\beta}_P(1+\alpha_R R)}{\eta(\beta+\bar{\beta}_P P)}(1-K) \approx 0$ is justified. Given this simplification, the new equation for phosphorylated paxillin at steady state is

$$0 = B\left(\frac{K^n}{L_K^n+K^n}\right)(1-P) - \delta_P P \Leftrightarrow P = B\frac{K^n}{\delta_P L_K^n+(\delta_P+B)K^n}. \quad \text{(S8)}$$

Using the result in (S8) and MATLAB tool *cftool*, we can fit this explicit expression for P to the data (see S4B Fig, right) and obtain estimates for the parameters B and L_K. Our results reveal that B = 4.26 s^{-1} and L_K = 5.77.

The parameter γ

The values of γ, the ratio of the total concentrations of PAK to Rac, and δ_R, the Rac inactivation rate, can be estimated from quantitative mass spectrometry (see subsection entitled "The concentrations of GIT, PIX, and Pax_{tot} for an overview of the method) and Rac inactivation assays, respectively. Using this approach, we obtain the estimates $\gamma = 0.003$ and $\delta_R = 0.009\ \mathrm{s}^{-1}$. The bifurcation diagram associated with these two values does not exhibit a monostable regime of uninduced states. In other words, the left saddle node is in the negative quadrant and the transition from the induced states to the uninduced state is not possible (results not shown). This appears to contradict the experimental observations in [14], showing that PAK inhibition by IPA-3 produces low Rac and high Rho activities. Based on this, we conclude that the above estimates are lower bounds for these two parameters and higher values must be chosen. To produce bistability with monostable regime of uninduced state, we choose $\gamma = 0.3$ and $\delta = 0.025\ \mathrm{s}^{-1}$ (see Fig 4).

Diffusion coefficients

The diffusion coefficients of active and inactive Rac1 are estimated from single molecule tracking of Rac1 [15], which showed that a subpopulation of Rac diffuses slowly while another diffuses quickly. The diffusion coefficients D_1 and D_2 of these two subpopulations were estimated in Table 1 of [15]. We assume here that D_1 is the slow-membrane bound diffusion coefficient associated with active Rac, and that D_2 is the fast diffusion coefficient associated with inactive Rac. Based on this, we conclude that $D_R = 0.437$ μm^2/s and $D_{R_i} = 0.020$ μm^2/s.

Furthermore, since Rac and Rho are similar in size, we assume that the same diffusion coefficients apply for active and inactive Rho, i.e. $D_\rho = 0.437$ μm^2/s and $D_{\rho_i} = 0.020$ μm^2/s. It is important to point out here that the estimates for diffusion coefficients used in [1,2] were not specific for Rac and Rho; that explains the discrepancy between their values and the ones presented here.

The diffusion coefficients of (phospho)paxillin were estimated in [16] using correlation spectroscopy, which showed that there are three subpopulations of paxillin, each possessing a distinct diffusion coefficient. Single point fluorescence correlation spectroscopy (FCS) revealed that diffusion coefficients ranged from ~0.02 μm^2/s to 1 μm^2/s in adhesions in CHO-K1 cells [16]. Similar analyses in MDA-MB-231 cells revealed that 80% of the diffusion coefficients in adhesions were slower than 0.03 μm^2/s [17]. Under the assumption that both phosphorylated and unphosphorylated paxillin reside within adhesions, we therefore estimate a relatively slow diffusion coefficient of 0.03 μm^2/s for both. Note, however, that polarization-like effects can occur for a range of values of D_{P_i} (from 0 to ~1 μm^2/s).

REFERENCES

1. Maree AFM, Jilkine A, Dawes A, Grieneisen VA, Edelstein-Keshet L. Polarization and Movement of Keratocytes: A Multiscale Modelling Approach. Bull Math Biol. 2006;

2. Jilkine A, Maree AFM, Edelstein-Keshet L. Mathematical Model for Spatial Segregation of the Rho-Family GTPases Based on Inhibitory Crosstalk. Bull Math Biol. 2007;

3. Reid T, Furuyashiki T, Watanabe G, Watanabe N, Chem JB, Reid T, et al. Cell Biology and Metabolism : Rhotekin , a New Putative Target for Rho Bearing Homology to a Serine / Threonine Kinase , PKN , and Rhophilin in the Rho-binding Domain Rhotekin , a New Putative Target for Rho Bearing Homology to a Serine / Threonine Kinase. 1996;271: 13556–13560.

4. Ren XD, Kiosses WB, Schwartz MA. Regulation of the small GTP-binding protein Rho by cell adhesion and the cytoskeleton. EMBO J. 1999;18: 578–585. doi:10.1093/emboj/18.3.578

5. Miyamoto Y, Reddig P, Juliano RL. Regulation of Signal Transduction by Integrins. Cell Adhesion. 2004. pp. 197–216.

6. Ren XD, Schwartz M a. Determination of GTP loading on Rho. Methods Enzymol. 2000;325: 264–272. doi:S0076-6879(00)25448-7 [pii]

7. Sander EE, Ten Klooster JP, Van Delft S, Van Der Kammen RA, Collard JG. Rac downregulates Rho activity: Reciprocal balance between both GTPases determines cellular morphology and migratory behavior. J Cell Biol. 1999;147: 1009–1021. doi:10.1083/jcb.147.5.1009

8. Schwanhausser B, Busse D, Li N, Dittmar G, Schuchhardt J, Wolf J, et al. Global quantification of mammalian gene expression control. Nature. 2011;

9. Schlenker O, Rittinger K. Structures of Dimeric GIT1 and Trimeric beta-PIX and Implications for GIT-PIX Complex Assembly. J Mol Biol. 2009; doi:10.1016/j.jmb.2008.12.050

10. Manser E, Loo T-H, Koh C-G, Zhao Z-S, Chen X-Q, Tan L, et al. PAK Kinases Are Directly Coupled to the PIX Family of Nucleotide Exchange Factors. Mol Cell. 1998;1: 183–192.

11. Schmalzigaug R, Garron ML, Roseman JT, Xing Y, Davidson CE, Arold ST, et al. GIT1 utilizes a focal adhesion targeting-homology domain to bind paxillin. Cell Signal. 2007; doi:10.1016/j.cellsig.2007.03.010

12. Zhang ZM, Simmerman JA, Guibao CD, Zheng JJ. GIT1 paxillin-binding domain is a four-helix bundle, and it binds to both paxillin LD2 and LD4 motifs. J Biol Chem. 2008;283: 18685–18693. doi:10.1074/jbc.M801274200

13. Slack BE. Tyrosine phosphorylation of paxillin and focal adhesion kinase by activation of muscarinic m3 receptors is dependent on integrin engagement by the extracellular matrix. Proc Natl Acad Sci U S A. 1997;

14. Byrne KM, Monsefi N, Dawson JC, Degasperi A, Bukowski-Wills JC, Volinsky N, et al. Bistability in the Rac1, PAK, and RhoA Signaling Network Drives Actin Cytoskeleton Dynamics and Cell Motility Switches. Cell Syst. 2016;2: 38–48. doi:10.1016/j.cels.2016.01.003

15. Das S, Yin T, Yang Q, Zhang J, Wu YI, Yu J. Single-molecule tracking of small GTPase

Rac1 uncovers spatial regulation of membrane translocation and mechanism for polarized signaling. Proc Natl Acad Sci. 2015;112: E267–E276. doi:10.1073/pnas.1409667112

16. Digman MA, Brown CM, Horwitz AR, Mantulin WW, Gratton E. Paxillin dynamics measured during adhesion assembly and disassembly by correlation spectroscopy. Biophys J. 2008;94: 2819–2831. doi:10.1529/biophysj.107.104984

17. Chiu C-L, Aguilar JS, Tsai CY, Wu G, Gratton E, Digman MMA, et al. Nanoimaging of focal adhesion dynamics in 3D. PLoS One. 2014;9: e99896. doi:10.1371/journal.pone.0099896
