# Supplementary figures and images for: Paxillin phosphorylation at serine 273 and its effects on Rac, Rho and adhesion dynamics
Source: PLoS Comput Biol. 2018 Jul 5;14(7):e1006303. doi: 10.1371/journal.pcbi.1006303 (PMC6053249; doi:10.1371/journal.pcbi.1006303)

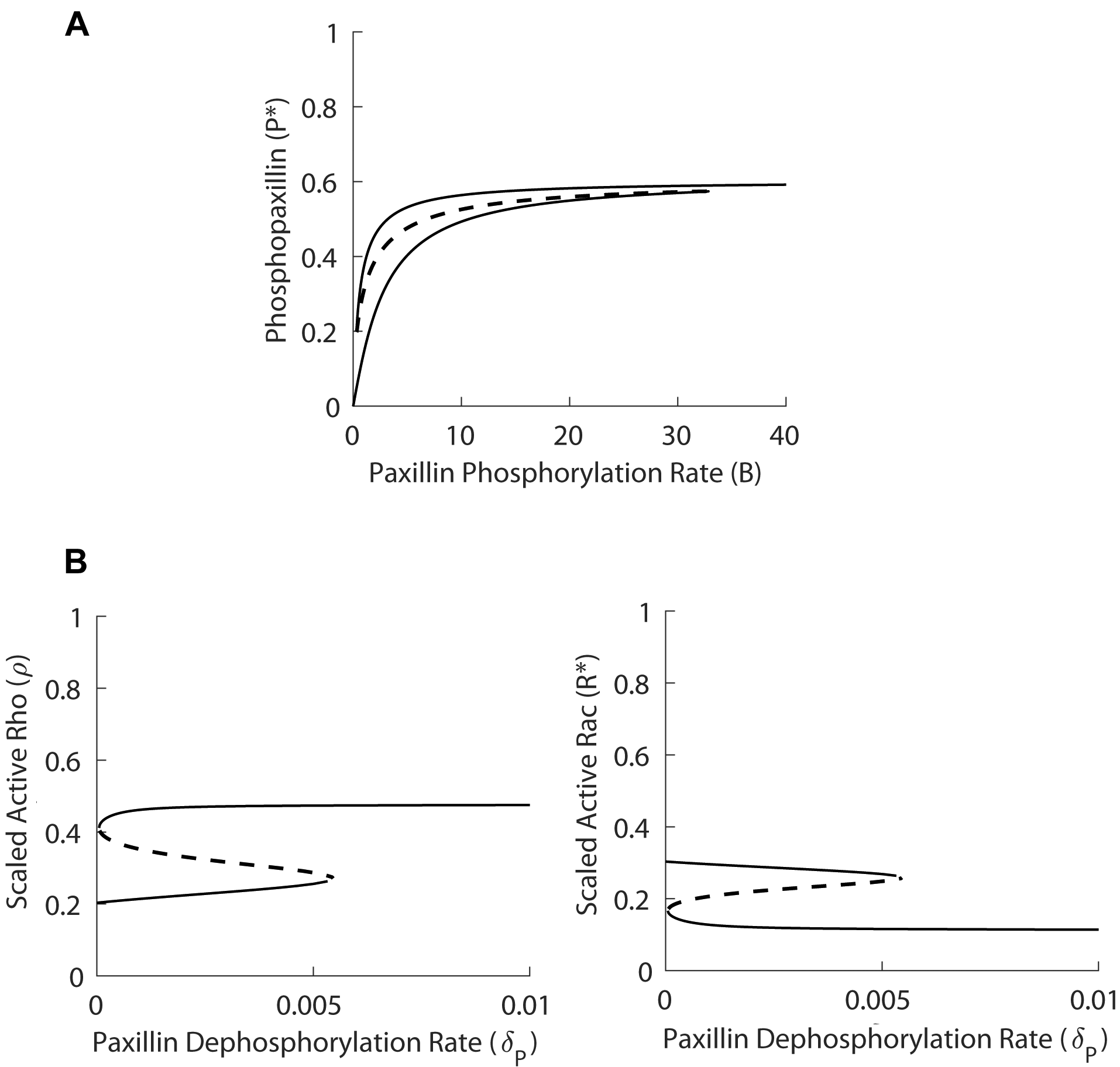

Supplement: S1 Fig — (A) Bifurcation diagram of phosphopaxillin P* with respect to the maximum paxillin-phosphorylation rate B, showing the steady state levels of P* in the induced (elevated R*) and uninduced (elevated ρ) states; solid lines represent stable steady states, dashed lines represent saddle points. Notice the sigmoidal profile of the bifurcation diagram and the small difference between the levels of P* in the induced and uninduced states (as compared to the larger difference between the levels of ρ and R* in the two states). (B) Bifurcation diagrams of active Rho ρ (left), and active Rac R* (right panel) with respect to the paxillin dephosphorylation rate δP, showing the steady state levels of these two variables in the induced (elevated R*) and uninduced (elevated ρ) states; solid lines represent stable steady states; dashed lines represent saddle points. (TIF) [file pcbi.1006303.s002.tif]

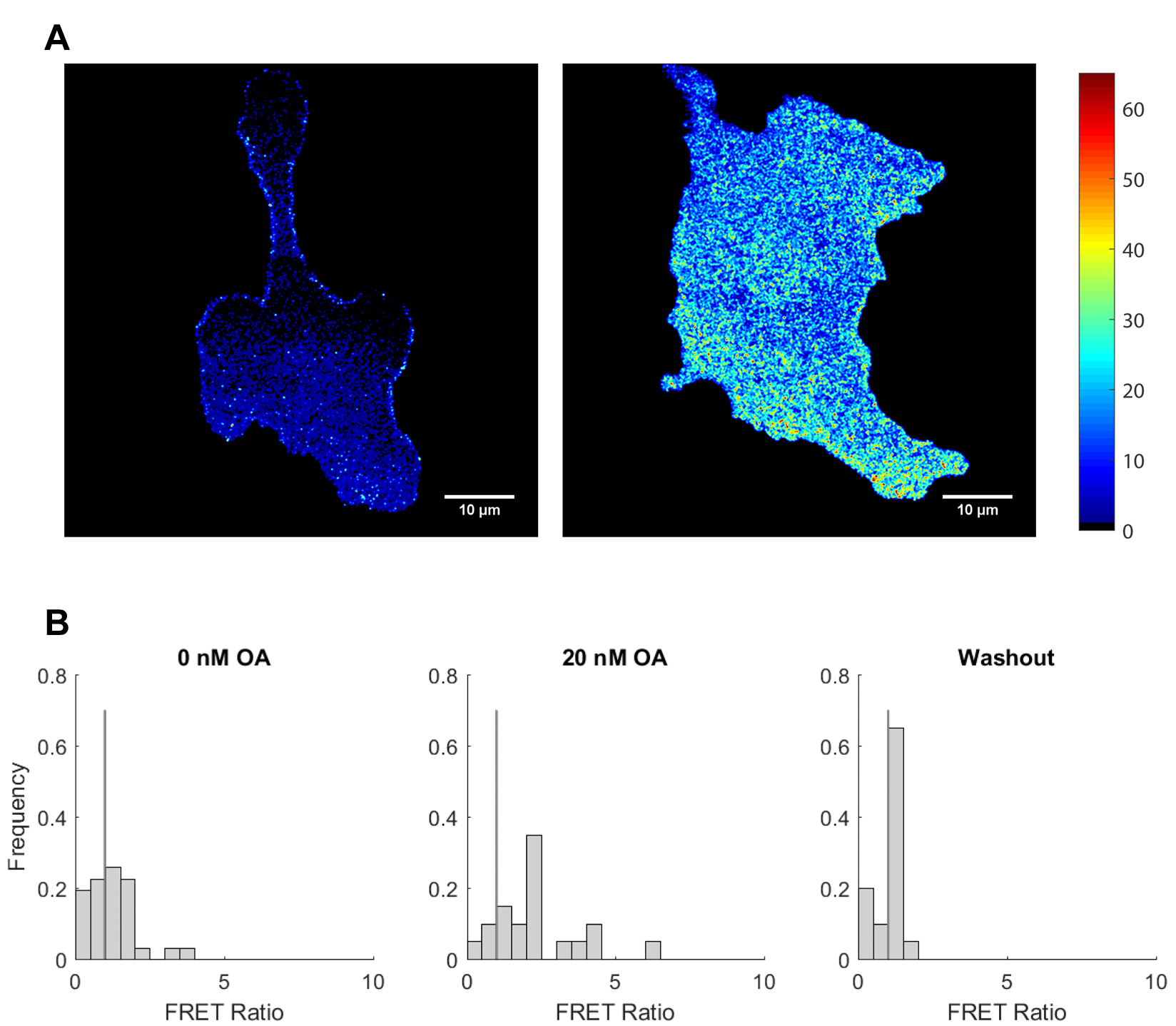

Supplement: S2 Fig — (A) Rac activity in CHO-K1 cells, quantified as a mean FRET ratio of the Rac1-2G biosensor across the entire cell, showing the variations in Rac activity ranging from low (left) to high (right); color bar, indicating magnitude of FRET ratio, shown on the right. The scale bar for both images is 5 μm. (B) The distributions of mean FRET ratios across multiple CHO-K1 cells (n = 20 − 31) with no treatment with okadaic acid (left), after 20 min of 20 nM treatment with okadaic acid (middle), and after 20 min of washout (right). The distribution shifts rightward with administration of okadaic acid, and the percentage of cells with high FRET ratios (>1, i.e. to the right of red line) increases from ~58% to ~85%, indicating an overall increase in Rac activity after inhibition of the paxillin dephosphorylation rate δP. After washout, the distribution shifts partially back to the left, with ~70% still exhibiting high FRET ratio. (TIF) [file pcbi.1006303.s003.tif]

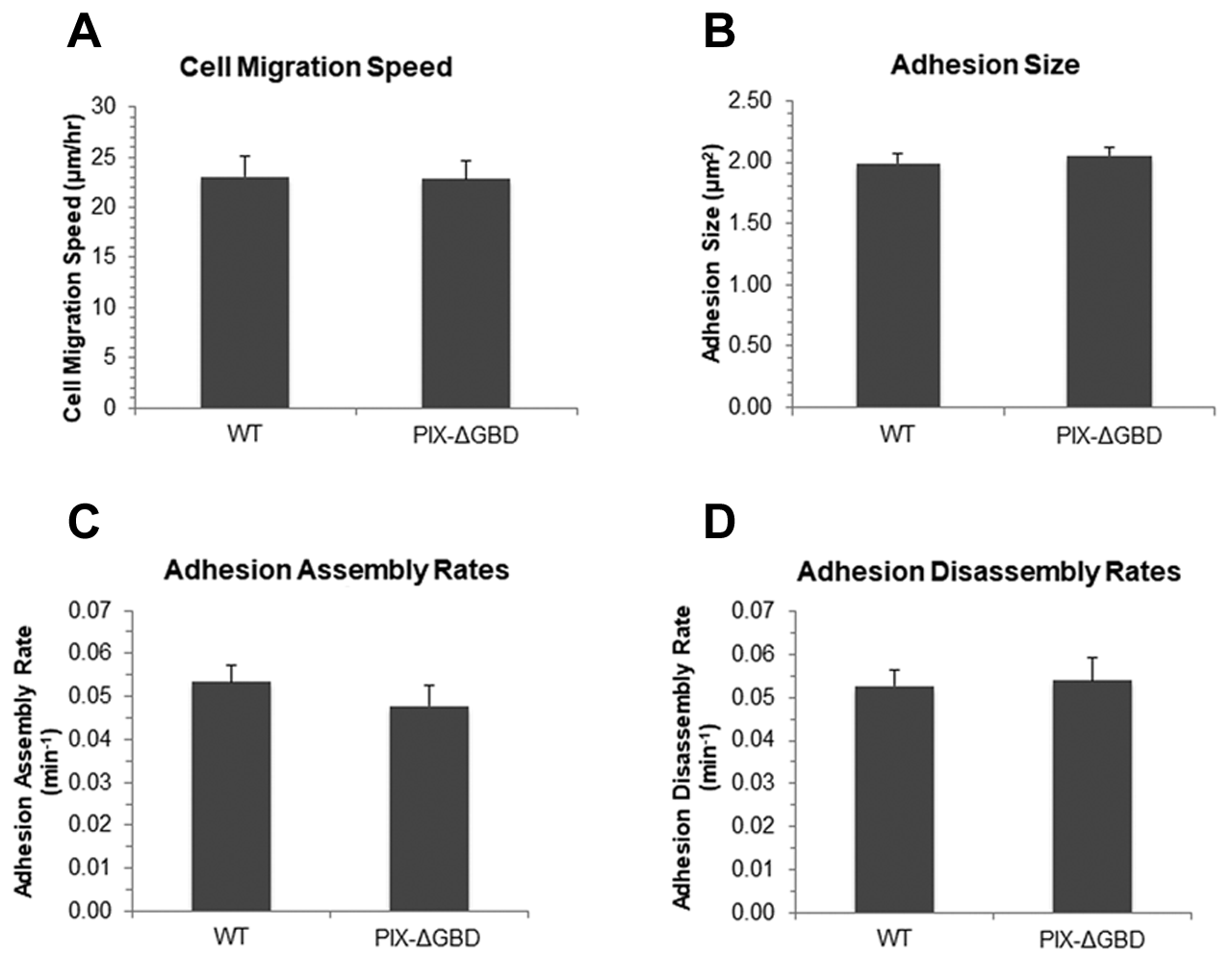

Supplement: S3 Fig — Transfecting CHO-K1 cells with GIT binding-deficient mutant of PIX (PIX-ΔGBD) does not alter migration velocity (A), average adhesion size (B) or adhesion assembly /disassembly rates (C and D, respectively) of these cells when compared to cells expressing wild type (WT) PIX. (TIF) [file pcbi.1006303.s004.tif]

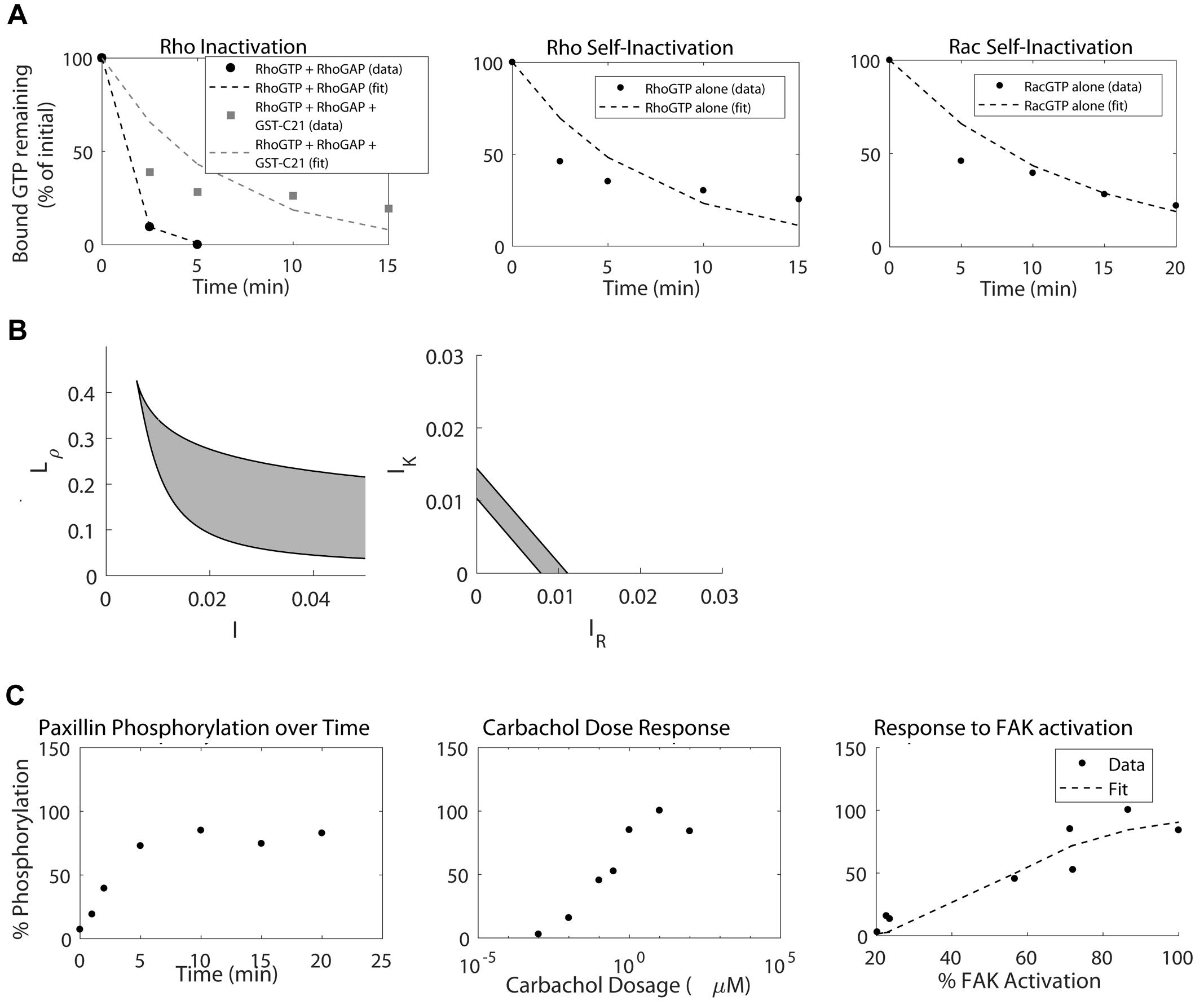

Supplement: S4 Fig — (A) Estimation of inactivation rates of Rho and Rac. (Left) Temporal profiles of Rho inactivation when incubated with RhoGAP alone (black dots) or with both RhoGAP and GST-C21 (grey squares). (Middle) Temporal profile of Rho self-inactivation (black dots). (Right) Temporal profile of Rac self-inactivation (black dots). Data points were digitized and fitted to mono-exponentially decaying functions (dashed lines) to calculate inactivation rates of Rho (under different conditions) and Rac. (B) Two-parameter bifurcation of the model with respect to Lρ and I (left panel) and IK and IR (right panel) used to provide upper bounds for Lρ, IR, and IK. The bistable regime (gray) lies between the two curves of saddle nodes, and the monostable regimes of induced and uninduced states lie above and below the bistable regime, respectively. (C) Estimation of B, LK, and δP. (Left) Temporal profile of paxillin phosphorylation following treatment with 100 μM of FAK activator carbachol (black dots). Using the steady state fraction of phosphorylated paxillin, the initial phosphorylation rate, as well as Assumptions (1)-(4), we estimated the paxillin dephosphorylation rate δP. (Middle) Dose response curve of paxillin phosphorylation when treated with varying concentrations of carbachol (black dots). (Right) Paxillin phosphorylation with respect to FAK activation (black dots) determined by combining the data in Middle panel with Western blot quantification of FAK response to carbachol treatment. The data points in the Right panel were fitted to Eq. S8 (dashed line), using a non-linear least squares fitting, to estimate the parameters B and LK. (TIF) [file pcbi.1006303.s005.tif]
