# Supplementary material for: Paxillin phosphorylation at serine 273 and its effects on Rac, Rho and adhesion dynamics
Source: PLoS Comput Biol. 2018 Jul 5;14(7):e1006303. doi: 10.1371/journal.pcbi.1006303 (PMC6053249; doi:10.1371/journal.pcbi.1006303)
Supplement: S1 Table — Agents associated with the transition arrows (e.g., RhoGEF and RhoGAP in reaction 1) are catalysts for these reactions. (PDF) [file pcbi.1006303.s006.pdf]

**Description of the Reaction**	**Reaction Schemes**	**Reaction Number**
Rac/Rho activation and inactivation	$RhoGDP \underset{RhoGAP}{\overset{RhoGEF}{\rightleftarrows}} RhoGTP$	1
	$RacGDP \underset{RacGAP}{\overset{RacGEF}{\rightleftarrows}} RacGTP$	2
PAK activation and inactivation	$RacGTP + PAK \rightleftarrows PAK\text{-}RacGTP$	3
Paxillin phosphorylation	$Pax \underset{PP2A}{\overset{PAK-RacGTP}{\rightleftarrows}} Pax_p$<br>$Pax \underset{PP2A}{\overset{Pax_p\text{-}GIT\text{-}PIX\text{-}PAK\text{-}RacGTP}{\rightleftarrows}} Pax_p$	4
Assembly of the GIT-PIX-PAK complex	$GIT + PIX \rightleftarrows GIT\text{-}PIX$	5
	$GIT\text{-}PIX + PAK \rightleftarrows GIT\text{-}PIX\text{-}PAK$	6
	$PIX + PAK \rightleftarrows PIX\text{-}PAK$	7
	$GIT + PIX\text{-}PAK \rightleftarrows GIT\text{-}PIX\text{-}PAK$	8
Formation of the PIX-PAK-RacGTP complex	$PIX\text{-}PAK + RacGTP \rightleftarrows PIX\text{-}PAK\text{-}RacGTP$	9
	$PIX + PAK\text{-}RacGTP \rightleftarrows PIX\text{-}PAK\text{-}RacGTP$	10
Paxillin-GIT-PIX-PAK binding	$Pax_p + GIT\text{-}PIX\text{-}PAK \rightleftarrows Pax_p\text{-}GIT\text{-}PIX\text{-}PAK$	11
Activation of Pax$_p$-bound PAK	$Pax_p\text{-}GIT\text{-}PIX\text{-}PAK + RacGTP \rightleftarrows Pax_p\text{-}GIT\text{-}PIX\text{-}PAK\text{-}RacGTP$	12
