# Supplementary material for: Paxillin phosphorylation at serine 273 and its effects on Rac, Rho and adhesion dynamics
Source: PLoS Comput Biol. 2018 Jul 5;14(7):e1006303. doi: 10.1371/journal.pcbi.1006303 (PMC6053249; doi:10.1371/journal.pcbi.1006303)
Supplement: S2 Table — (PDF) [file pcbi.1006303.s007.pdf]

**Carbachol Dosage (µM)**	0	0.001	0.01	0.1	0.3	1	10	100
**Percentage Active FAK**	23.6	20.3	22.7	56.6	72.0	71.3	86.6	100
